# DNA damage response signaling: A common link between cancer and cardiovascular diseases

**DOI:** 10.1002/cam4.5274

**Published:** 2022-09-26

**Authors:** Sepideh Nikfarjam, Krishna K. Singh

**Affiliations:** ^1^ Department of Anatomy and Cell Biology, Schulich School of Medicine and Dentistry University of Western Ontario London ON Canada; ^2^ Department of Medical Biophysics, Schulich School of Medicine and Dentistry University of Western Ontario London ON Canada

**Keywords:** DNA damage repair, cancer, cardiovascular disease, oxidative stress

## Abstract

DNA damage response (DDR) signaling ensures genomic and proteomic homeostasis to maintain a healthy genome. Dysregulation either in the form of down‐ or upregulation in the DDR pathways correlates with various pathophysiological states, including cancer and cardiovascular diseases (CVDs). Impaired DDR is studied as a signature mechanism for cancer; however, it also plays a role in ischemia‐reperfusion injury (IRI), inflammation, cardiovascular function, and aging, demonstrating a complex and intriguing relationship between cancer and pathophysiology of CVDs. Accordingly, there are increasing number of reports indicating higher incidences of CVDs in cancer patients. In the present review, we thoroughly discuss (1) different DDR pathways, (2) the functional cross talk among different DDR mechanisms, (3) the role of DDR in cancer, (4) the commonalities and differences of DDR between cancer and CVDs, (5) the role of DDR in pathophysiology of CVDs, (6) interventional strategies for targeting genomic instability in CVDs, and (7) future perspective.

## 1 INTRODUCTION

**FIGURE 1:**
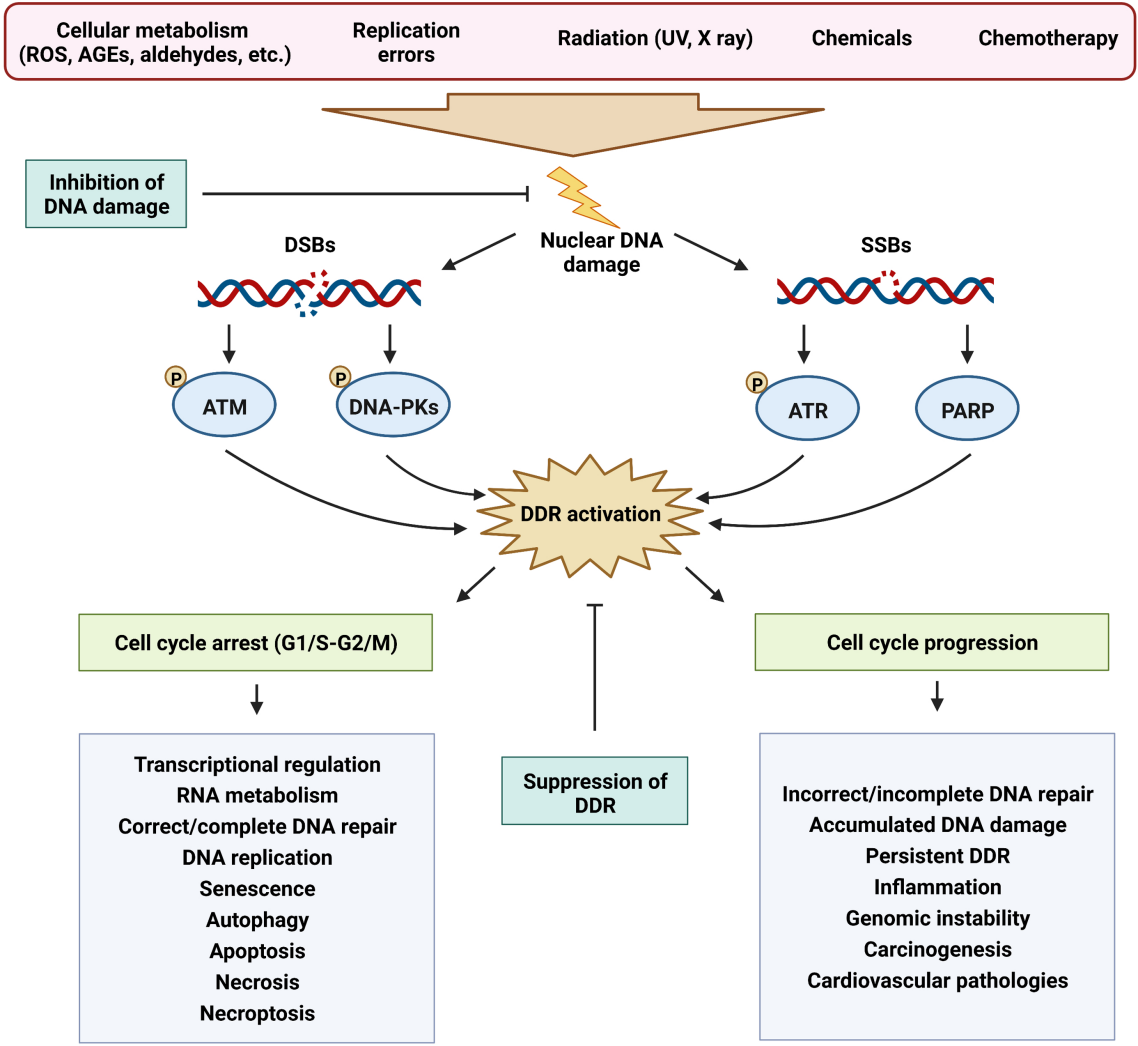
DNA damage response signaling in health and disease and potential interventional strategies targeting DNA damage. DNA damage can be initiated by a variety of endogenous (cellular metabolism and replication errors) and exogenous (radiation, chemicals, and chemotherapeutic agents) factors. Upon detection of DNA damage by damage sensors, DNA damage response signaling is orchestrated by four main upstream regulators, namely ATM and DNA‐PKs (mediating DSB repair), as well as ATR and PARP (regulating SSB repair). Normally, cells undergo a transient cycle arrest either at the G1/S or G2/M phase to be able to confront the damaged DNA. Subsequently, a variety of precisely regulated transcriptional and metabolic modifications eventually lead to successful DNA damage repair and continuing DNA replication or cell cycle reactivation. However, cells can undergo permanent cell cycle arrest (senescence), cell death (apoptosis, necrosis, or necroptosis), or autophagy if DNA damage is not properly or completely repaired. On the other hand, the inability to activate cell cycle checkpoints in a timely manner can result in incorrect or incomplete DNA repair, accumulated DNA damage, persistent DDR, and genomic instability, all of which are central mechanisms orchestrating carcinogenesis as well as a variety of cardiovascular pathologies. In general, therapeutic strategies targeting DNA damage and DDR mechanisms can be classified into two main categories: (i) therapies that prevent DNA damage and (ii) strategies that suppress the activation of DDR mechanisms. AGEs, advanced glycation end products; ATM, ataxia‐telangiectasia‐mutated kinase; ATR, ATM‐ and RAD3‐related kinase; DDR, DNA damage response; DNA‐PKs, DNA‐dependent protein kinases; DSBs, DNA double‐strand breaks; PARP, poly(ADP‐ribose) polymerase; ROS, reactive oxygen species; SSBs, DNA single‐strand breaks.

Genome integrity is constantly endangered by thousands of factors that are capable of inducing DNA damage each day.^1^ DNA lesions can arise from either endogenous normal metabolic processes or exogenous physical and chemical factors. Endogenous cellular processes include the production of reactive oxygen species (ROS) that can trigger oxidative base lesions and DNA mismatch errors caused by DNA polymerase enzymes during replication.^2^ Exogenous factors include ionizing and ultraviolet (UV) radiation that can trigger the formation of roughly 10^5^ DNA lesions such as^3^, ^4^ photoproducts and pyrimidine dimers per cell everyday.^5^ Ionizing radiation in sunlight or therapeutic radiation can instigate single‐strand breaks (SSBs) and double‐strand breaks (DSBs) in the DNA backbone. Chemical factors including smoke, vehicle exhaust and factory fumes, and chemotherapy agents, such as bleomycin and cisplatin, can also give rise to DNA damage by hindering the DNA topoisomerase enzyme activity or adding alkyl groups to bases. Other factors, such as infection by microorganisms can also lead to DNA damage.^3^ The accurate and rapid restoration of DNA following damage is pivotal for cell survival and for preserving genomic integrity. Genomic instability caused by inaccurate or defective DNA repair can negatively impact the genetic code and lead to the accumulation of mutations, which eventually increases the incidence of carcinogenesis.^6^ Accordingly, cells have evolved intricate mechanisms to confront and repair DNA lesions, collectively known as the DNA damage response (DDR) signaling. DDR involves a coordinated network of proteins, including sensors, transducers, and effectors, which altogether create a complex signaling cascade to respond to genotoxic stress. As summarized in Figure 1, the DDR system is mainly conducted by the phosphoinositide 3 kinase proteins (PI3Ks): ataxia‐telangiectasia‐mutated (ATM), ATM‐ and RAD3‐related (ATR), and DNA‐dependent protein kinase (DNA‐PK) and by the poly (ADP‐ribose) polymerase (PARP) proteins. DNA‐PK and ATM mainly mediate the detection and repair of DSBs,^6^ whereas PARP1 and ATR are activated by SSB lesions created at DSB sites or collapsed replication forks.^6^, ^7^

Normally, once the DDR cascade is initiated, cells undergo transient cell cycle arrest to repair the damaged DNA, a process ending in proper DNA repair and DDR inactivation.^8^ However, excessive or persistent DNA damage or DDR activation can result in accumulated DNA damage and trigger cell death, senescence, or tumorigenesis.^5^ DDR activation may exert differential effects depending on the cell type. The cardiovascular system consists of multiple cell types, including fibroblasts, vascular smooth muscle cells (VSMCs), cardiomyocytes, immune, progenitor, epithelial, and endothelial cells. A growing number of *in vitro* and *in vivo* studies have demonstrated the presence of DNA damage in nearly all cell types of the cardiovascular system, and genomic instability has been suggested as a potential factor in the development and/or progression of CVDs although remaining greatly underexplored. The purpose of this review is to thoroughly discuss different DDR mechanisms, their functional cross talk, the role of DDR in malignancies as well as CVDs, the common features and differences of DDR in cancer and CVDs, as well as therapeutic strategies targeting DNA damage and DDR in CVDs.

## 2 DNA DAMAGE RESPONSE SIGNALING PATHWAYS

**FIGURE 2:**
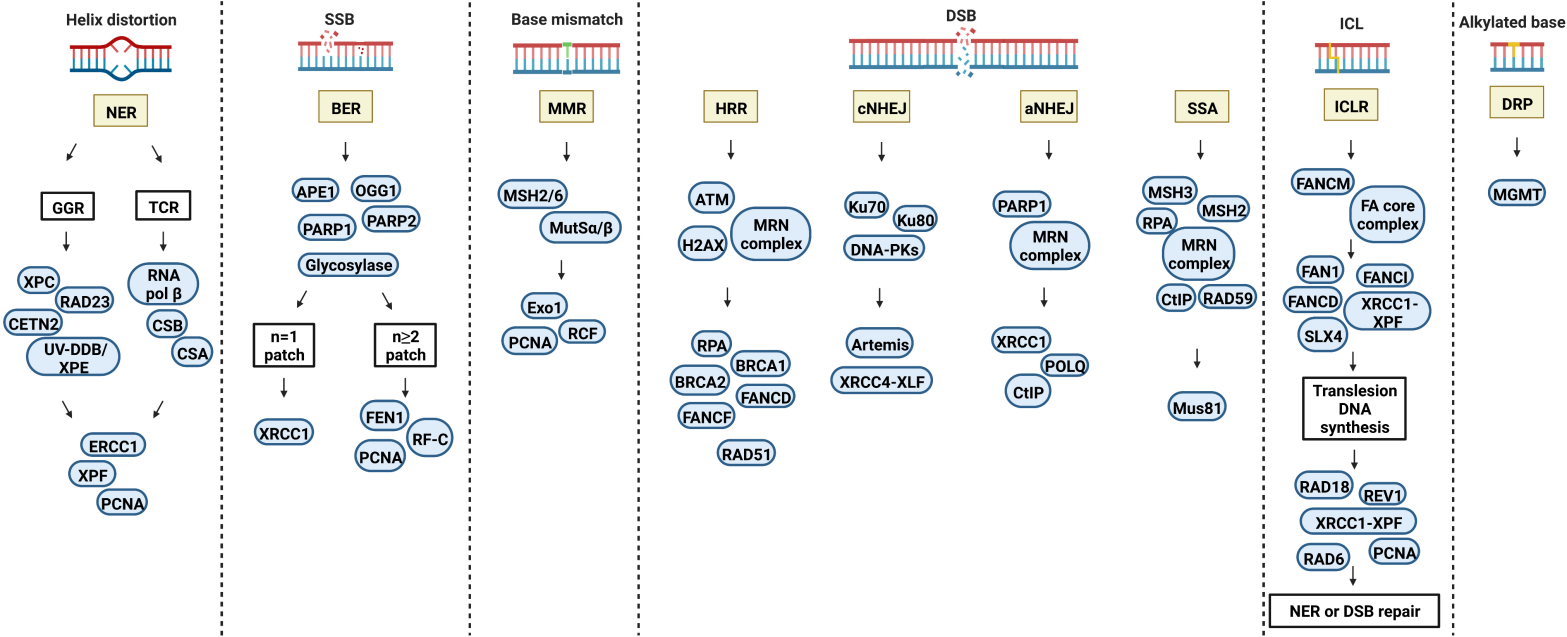
DNA damage response signaling pathways. The DDR network generally consists of six main pathways that are activated depending on the type and site of DNA damage. aNHEJ, alternative nonhomologous end joining; APE1, apurinic/apyrimidinic endonuclease 1/redox factor 1; ATM, ataxia‐telangiectasia‐mutated kinase; BER, base excision repair; cNHEJ, canonical nonhomologous end joining; DNA‐PK, DNA‐dependent protein kinase; DRP, direct repair pathway; ERCC1, excision repair cross‐complementing protein 1; Exo1, 5′ exonuclease 1; FEN1, Flap Endonuclease 1; GGR, global genome repair; HRR, homologous recombination repair; MGMT, O6‐methylguanine‐DNA methyltransferase; MMR, mismatch repair; MSH2, mutator S homolog 2; MutSα, mutator Sα; NER, nucleotide excision repair; PARP, poly(ADP‐ribose) polymerase; ICLR, interstrand crosslink repair; SSA, single‐strand annealing; SSB, single‐strand break; TCR, transcription‐coupled repair.

The DDR network generally includes the following six different pathways as briefly summarized in Figure 2.

### 2.1 Single‐strand break repair

#### 2.1.1 Nucleotide excision repair (NER)

Large DNA lesions that disturb the structure of the DNA helix are recognized and removed by NER.^9^ NER involves the removal of DNA adducts produced by UV radiation (cyclobutane pyrimidine dimers), 6‐4 photoproducts, or DNA lesions generated by ROS, environmental carcinogens (benzo[a]pyrene), or genotoxic drugs (cisplatin and melphalan).^10^, ^11^ NER can be categorized into two sub‐pathways (Figure 2) such as global genome repair (GGR) and transcription‐coupled repair (TCR). The only difference between these sub‐pathways is in the recognition of DNA damage. In GGR, helix distortion caused by DNA adduct initiates the employment of the damage detection factor XPC/RAD23/CETN2 and UV‐DDB, whereas, in TCR, detection of damage is induced by a stalled RNA polymerase β upon a DNA damage site which is then removed by Cockayne syndrome A (CSA) and B (CSB) proteins to facilitate the accessibility of the lesion for NER proteins. After the detection of damage, both GGR and TCR progress via common pathways. Defective NER can result in several human genetic disorders associated with photosensitivity.^12^

#### 2.1.2 Base excision repair (BER)

BER mediates the recognition and removal of damaged DNA bases, which are not typically helix‐distorting.^13^ DNA lesions recognized by BER are created by spontaneous hydroxylation or deamination of bases, through oxidation of nucleotides by ROS produced as the result of either normal metabolism or environmental stresses (ionizing radiation, oxidizing chemicals, or smoking)^14^ or by alkylation of bases produced via endogenous or exogenous elements (antineoplastic drugs and carcinogens), all of which are able to trigger mutations if left unrepaired.^15^ There are two sub‐pathways of BER, termed as short‐patch or single‐nucleotide and long‐patch (Figure 2). The activation of each sub‐pathway is dependent on the source and nature of DNA damage, the nature of the abasic (apurinic/apyrimidinic) site, and the ongoing cell cycle phase. The short‐patch route handles single‐base lesions in the G1 phase. On the other hand, the long‐patch route is involved in the resynthesis of larger lesions (2–8 nucleotides neighboring the apurinic/apyrimidinic site) during S or G2. DNA glycosylases are among the most important BER enzymes that perform the hydrolyzation of the N‐glycosylic bond between the sugar‐phosphate backbone and the damaged base, generating an apurinic/apyrimidinic intermediate site.^15^

#### 2.1.3 Mismatch repair (MMR)

MMR is the major mechanism for the preservation of replication fidelity. MMR handles the removal of base insertion/deletion and substitution mismatches caused by replication errors evading the proofreading activity of DNA polymerases.^16^ DNA lesions are recognized by Mutator Sα (MutSα), a heterodimer complex consisting of the DNA mismatch repair proteins Mutator S homolog 2 (MSH2), and MSH6. MutSβ is another heterodimer complex that contains MSH2 and MSH3 and binds only to insertion/deletion mismatches. Upon damage recognition, Mutator Lα (MutLα) [MLH1/postmeiotic segregation increased 2 (PMS2)] or MutLβ (MLH1/MLH3) endonucleases cut DNA near the damage site. The enzyme 5′ exonuclease 1 (Exo1) enters the damage site through the nick, then degrades DNA past the mismatch. The resultant ssDNA gap is filled in and sealed by polymerase δ and DNA ligase I, respectively.^17^, ^18^ Defects in MMR can cause microsatellite instability and is related to certain prognosis, clinical features, therapy response, and immune checkpoint blockade.^19^, ^20^

### 2.2 Double‐strand break repair

DSBs can be the product of both exogenous factors, such as genotoxic drugs and ionizing radiation,^7^ and endogenous events, such as replication fork collapse, oxidative stress, and telomere erosion.^21^ DSBs can also occur as a part of programmed cellular events during meiosis, V(D)J recombination, and class‐switch recombination.^22^ However, unrepaired DSBs are severely dangerous for cells as they can lead to mutations, chromosomal abnormalities, and cell death.^23^ Cells have developed the following mechanisms to repair DSBs (Figure 2).

#### 2.2.1 Homologous recombination repair (HRR)

HRR is a major contributor to error‐free DDR that acts during the S and G2 stages of the cell cycle to locate a large homologous DNA sequence on a sister chromatid that can be utilized as a template for resynthesizing damaged or missing bases.^24^ DSBs are recognized by histone H2AX, the mediator of DNA damage checkpoint protein 1 (MDC1), and RING finger protein 8 (RNF8). Upon activation of mediator proteins including Rap80, Abraxas, and BRCA1, 5ʹ‐3ʹ DNA end resection is performed by the MRN complex, which includes MRE11–RAD50–NBS1 (Nijmegen breakage syndrome protein 1), to generate a 3ʹ ssDNA tail. The MRN complex also binds to the BRCA1–CtBP‐interacting protein (CtIP) complex which is necessary to activate the DNA damage signaling kinases ATM, ATR, and CHK2.^25^, ^26^, ^27^ The ssDNA is then coated with the replication protein A (RPA) to prevent it from binding to other ssDNA molecules in the form of spurious secondary structures. RPA also serves as a platform for the loading of RAD51 recombinase and must be removed by recombination mediators to enable RAD51 filament formation.^28^ The formation of BRCA1–partner and localizer of BRCA2 (PALB2)–BRCA2 complex is required for RAD51‐dependent HRR. There are three sub‐pathways of HRR, activation of which is dependent on how two DNA ends interact at the recombination synapse and operate on the D‐loop created after the formation of synapsis. The major repair pathway in somatic cells is the noncrossover synthesis‐dependent strand annealing. The creation of a double Holliday junction intermediate can result in crossing over in meiotic cells. If cells fail to involve the second end of the break or fail to replace the nascent strand, abnormal replicative HRR responses including long tract gene conversion and break‐induced replication will occur. BRCA2 acts as the major mediator of recombination in vertebrates and in several fungal species.^29^, ^30^, ^31^ BRCA2, which is constitutively bound to the proteasomal component DSS1, interacts with both ssDNA and RAD51 monomers and with BRCA1–BARD1 via the PALB2 protein.^32^

#### 2.2.2 Canonical nonhomologous end joining (cNHEJ)

cNHEJ is an error‐prone DNA repair mechanism that operates during the entire cell cycle and entails the interaction of the Ku70–Ku80 (also known as XRCC6–XRCC5) heterodimer to DNA ends at DSBs. cNHEJ involves a two‐step mechanism of DNA end synapsis.^33^ First, a long‐range synapsis is established by Ku70–Ku80 and DNA‐PKs; then, the two ends become closely associated via XLF, XRCC4–ligase IV, and DNA‐PKs kinase activity.^34^ Ku facilitates recruitment of other cNHEJ proteins such as the DNA‐dependent protein kinase catalytic subunit (DNA‐PKs), DNA ligase IV, and the associated scaffolding factors such as XRCC4, XRCC4‐like factor (XLF), and paralog of XRCC4, and XLF (PAXX).^35^, ^36^, ^37^, ^38^, ^39^ DNA‐PKs activate the XRCC4–ligase IV complex to religate the broken DNA ends. However, prior to binding, the MRN complex, the Flap Endonuclease 1 (FEN1), and Artemis operate together to process DSB ends.^40^, ^41^ Deficiencies in cNHEJ are associated with genomic rearrangements and chromosomal translocations,^42^ defective V(D)J recombination, and immune defects.^43^

#### 2.2.3 Alternative nonhomologous end joining (aNHEJ)

aNHEJ, also referred to as microhomology‐mediated end‐joining, is another pathway of DSB repair.^44^ The most outstanding feature of aNHEJ is the utilization of 5–25 bp microhomologous sequences during the alignment of DSB ends prior to religation, thus giving rise to deletions that flank the original break.^45^ aNHEJ is often related to pathogenic chromosomal errors such as inversions, deletions, and translocations.^46^, ^47^

Complex regulatory mechanisms regulate the choice between the HRR and cNHEJ pathways. These mechanisms mainly involve competition between BRCA1, which favors HRR, and the p53‐binding protein 1 (53BP1), which promotes cNHEJ. Histone H4 methylation by Multiple Myeloma SET (MMSET) leads to the employment of 53BP1 at the DSBs, preventing DNA end resection by the MRN complex, C‐terminal binding protein 1 interacting protein (CtIP), and BRCA1. On the other side, acetylation of histone H4 by Tip60 (Tat‐interactive protein) prevents 53BP1 employment while promoting BRCA1 occupancy and HRR. Cell cycle regulatory proteins including the cyclin‐dependent kinases are also important in determining the pathway of choice to repair DSBs.^22^

#### 2.2.4 Single‐strand annealing (SSA)

SSA is a very efficient yet highly mutagenic mechanism to resolve DSBs.^22^, ^49^ Following a DSB between homologous repeats, DNA end resection creates 3′ ssDNA tails, giving rise to flanking homologous sequences which are annealed together to create a synapsedintermediate. Ligation is later performed by endonucleolytic cleaving of nonhomologous 3′ ssDNA tails and subsequent gap filling by a polymerase. SSA is genetically different from other HRR mechanisms since it operates independently of RAD51 and is dependent on the RAD52 paralog, RAD59, instead. SSA is essential to repair chromosomal DSBs that have undergone extensive end resection but cannot be restored by HRR or aNHEJ.^50^ However, SSA can be relatively mutagenic leading to rearrangement between repeat elements. The importance of the SSA repair mechanism is dependent on several factors, such as the cell cycle phase, the presence or absence of the sister chromatid, and the length of uninterrupted homology.^50^

#### 2.2.5 Interstrand cross‐link repair (ICLR)

DNA interstrand cross links can be formed by exogenous chemicals, such as cyclophosphamide, cisplatin, melphalan, psoralen, and mitomycin C,^50^ or endogenously produced aldehydes.^51^ Such lesions are considered critical because if left unrepaired, they can give rise to replication or cell cycle arrest and eventually cell death.^52^ Mammalian cells have developed three pathways for the detection of cross‐links. ICLR adducts can be detected through disturbed DNA duplex by damage‐recognizing factors, via interacting with the transcription machinery, and stalled replication fork, all of which trigger a repair response. In nonproliferating cells, ICLR is performed by NER and by the FANCM DNA translocase that enables nuclease entrance to the damage site. Interestingly, ICLR is integrated into the DNA replication system in S‐phase cells and is dependent on HRR.^53^ In human cells, there are four distinct steps to accomplish ICLR: (i) the interstrand cross‐link is unhooked on one strand, and a DSB is induced in a DNA replication‐dependent manner, (ii) translesion DNA synthesis is performed by utilizing the unhooked interstrand cross‐link as a template, (iii) DSB is processed and the collapsed DNA replication fork is restored, and (iv) the residual unhooked interstrand cross‐link is removed.^54^ Defects in proteins of the ICLR pathway play central roles in the pathophysiology of Fanconi anemia,^55^ Cockayne syndrome,^56^ trichothiodystrophy,^57^ xeroderma pigmentosum,^58^ and cerebro‐oculo‐facio‐skeletal syndrome.^59^

#### 2.2.6 Direct repair pathway (DRP)

The most distinguishing property of the DRP is that it is a single‐step route with only one protein, O6‐methylguanine‐DNA methyltransferase (MGMT), handling the process.^61^ The DRP is involved in the removal of aberrant alkyl groups caused by alkylating agents including procarbazine, dacarbazine, and temozolomide. MGMT removes alkyl groups from guanine or thymine and transfers them to a cysteine residue of MGMT.^62^ The alkylated MGMT is functionally deactivated and eliminated by the ubiquitin proteolysis pathway. If left unrepaired, alkyl adducts can lead to thymine mismatch during replication, causing G:C to A:T transitions or strand breaks.^63^

## 3 THE FUNCTIONAL CROSS TALK AMONG DISTINCT DNA DAMAGE RESPONSE PATHWAYS

**FIGURE 3:**
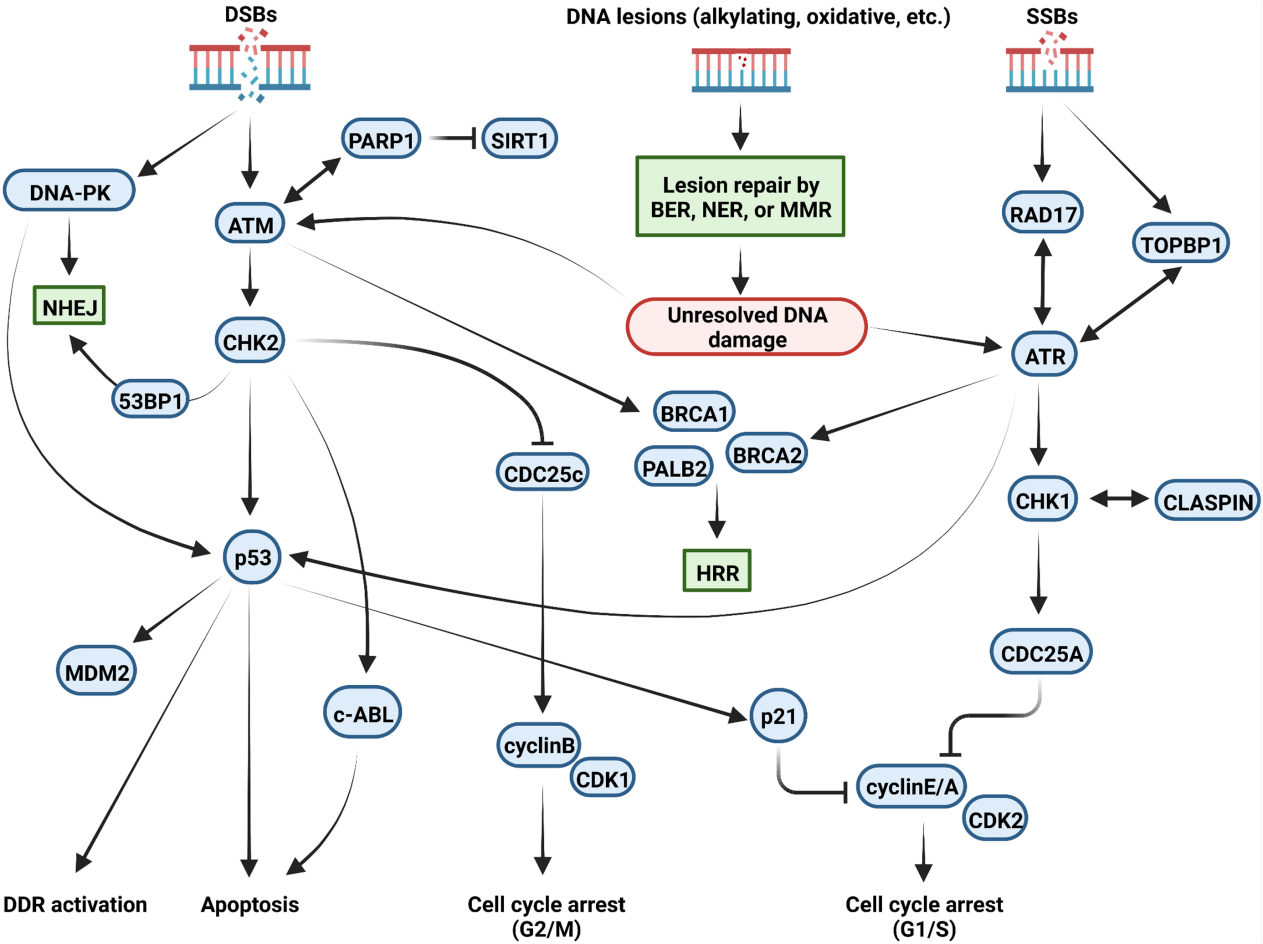
Functional cross talk among different DNA damage response mechanisms. Upon identification of DNA lesions (such as oxidative, alkylating lesions, etc.), the damaged sites are immediately restored through one of the lesion repair pathways: BER, NER, or MMR. In case of unresolved DNA lesions or the presence of DNA strand breaks, DDR signals are orchestrated via four main upstream regulators: ATM and DNA‐PKs, ATR, and PARP1. These proteins amplify the DDR signals via downstream regulators such as CHK1 and CHK2 and transduce the signals to downstream effectors including p53 and p21. The systematic function of complex DDR network sensors, transducers, and effectors will eventually lead to cell cycle arrest (G1/S or G2/M), selection of a particular DDR mechanism (HRR or NHEJ; based on the type and site of DNA damage), and cell fate decisions (apoptosis). ATM, ataxia‐telangiectasia‐mutated kinase; ATR, ATM‐ and RAD3‐related kinase; BER, base excision repair; DDR, DNA damage response; DNA‐PKs, DNA‐dependent protein kinases; DSBs, DNA double‐strand breaks; DDR, DNA damage response; MMR, mismatch repair; NHEJ, nonhomologous end joining; NER, nucleotide excision repair; PARP, poly(ADP‐ribose) polymerase; SSBs, DNA single‐strand breaks.

The DDR pathways evaluate the scale and severity of DNA damage and trigger cell cycle arrest, repair, senescence, or apoptosis in the case of irreversible damage. If repair is initiated, one of the DDR routes mentioned above will be activated. Based on the type and extent of the damage, there is significant cross talk between the various DDR mechanisms (Figure 3). The detectors of DDR signaling activate mediators and effectors by employing them at the DNA damage site. The ATM pathway operates throughout the entire cell cycle. Following DSB formation, the break site is recognized by the MRN sensor complex which employs the transducer kinase ATM at the break site.^28^, ^64^ ATM activation then results in the phosphorylation of H2AX as well as its ubiquitination by RNF8 and RNF168, resulting in the employment of downstream effectors BRCA1, BRCA2, and 53BP1.^65^, ^66^ In addition, the phosphorylated H2AX, known as γH2AX, facilitates the interaction of MDC1 at the damage site, where it is later phosphorylated by ATM.^67^ This precedes the phosphorylation and activation of cell cycle checkpoint proteins such as CHK2 and p53 that trigger cell cycle arrest to inhibit replication of damaged and/or unrepaired DNA.^6^ The tumor suppressor protein p53 is a critical sensor of DNA damage that functions downstream to ATM/ATR/DNA‐PK and plays a central role in the maintenance of genome integrity by deciding between the dilemma of cell cycle arrest or apoptosis.

On the other hand, ATR is activated at ssDNA regions formed at DSB overhangs or collapsed replication forks^6^ and acts in an ATM‐dependent manner in the S and G2 phases of the cell cycle.^68^, ^69^ Indeed, it is now well known that the ATR pathway is also capable of activating downstream elements of the ATM arm.^70^ Upon replication fork collapse or UV treatment, ssDNA regions are quickly coated by RPA. This leads to the recruitment of ATR to the site of DNA damage via interacting with ATR‐interacting protein (ATRIP).^71^ Several protein complexes such as the RAD17‐replication factor C 2 (RFC2) clamp loader complex and the RAD9‐RAD1‐Hus1 (9‐1‐1 complex) are serially employed in the RPA‐coated ssDNA, which eventually leads to the localization of topoisomerase II binding protein 1 (TOPBP1). The interactions of TOPBP1 with ATRIP and RAD of the 9‐1‐1 complex result in ATR activation,^72^ which precedes the phosphorylation of p53 and CHK1 effector proteins, cell cycle arrest, and DNA repair.^72^ Unlike the ATM and ATR kinases, DNA‐PK is involved in the DSB repair via the NHEJ pathway. DNA‐alkylating agents are capable of activating both ATM and ATR pathways. Although these agents inflict stress on advancing replication forks, they can also trigger strand breaks in some cases.^73^

## 4 DNA DAMAGE RESPONSE SIGNALING IN CANCER

In the section above, the essential role of the various DRS mechanisms in guarding the genome integrity is thoroughly explained. It is not surprising that deficiencies in any of these pathways would be deleterious for cell physiology. Aberrant operation or overburdening of DDR mechanisms and/or checkpoints can lead to the accumulation of mutations, which may initiate a series of events that finally lead to genomic instability and malignant transformation (Figure 1). In order to provide a comprehensive signature of aberrations associated with carcinogenesis, more than 9000 samples, including 33 different cancer types, were investigated by the Cancer Genome Project and Cancer Genome Atlas. These mutations can be generally classified as “driver,” which directly triggers tumor formation and development, and “passenger,” promoting cancer development as a consequence of their accumulation.^74^ In about a third of the cases, somatic mutations associated with DDR genes were observed. One example is the MLH1 and MSH2 mutations that result in dysfunctional MMR and thereby defective recognition and restoration of replicative lesions on DNA which eventually prime malignant outcomes^75^ or predisposition to cancer.^76^ Due to the central role played by DDR in malignant transformation and the associated consequences, anticancer therapies mostly target DDR either directly or indirectly.^77^, ^78^ There is a high probability that different DNA damage thresholds exist at distinct stages of tumor progression.^79^ The DDR pathway is quickly primed and precisely controlled in tumor cells as in normal cells, which suggests the possibility of targeting certain steps and proteins of the DDR machinery to hinder tumor growth. However, DDR is a double‐edged sword in the context of cancer development and therapy. The DDR contributes to tumor cell protection and survival as it repairs their restorable DNA damages, also when they are primed by DNA‐targeted therapies. This event is a major pathway in generating resistance to a genotoxic intervention. Any missing or defective canonical pathways of DDR can result in the dysregulation of DNA restoration routes leading to genomic instability which is a significant hallmark of cancer. Aberrant mechanisms may be finally compensated to substitute DDR routes creating an environment that highly promotes tumorigenesis and resistance to genotoxic treatments.^80^ DDR in cancer is reviewed in detail elsewhere.^81^

## 5 DNA DAMAGE RESPONSE SIGNALING: COMMONALITIES AND DIFFERENCES BETWEEN CANCER AND CARDIOVASCULAR DISEASES

### 5.1 DNA damage response in cardiovascular diseases

While originally considered two distinct diseases, recent investigations have revealed remarkable commonalities between CVDs and cancer, including common risk factors and intracellular pathways for disease development and progression. Of particular interest, many of the “hallmarks of cancer,” such as inflammation, genomic instability, cellular proliferation, cell death, therapy resistance, and angiogenesis represent the pathophysiologic routes common to both cancer and CVDs.^82^ A growing amount of clinical and preclinical evidence has revealed the presence of DNA damage and activation of DDR signaling in CVDs.^83^, ^84^, ^85^ While DDR activation was initially assumed to be a cell cycle arrest response limited to dividing cells, it became evident that DDR is also activated in postmitotic cells such as cardiomyocytes.^86^ Enhanced senescence and apoptosis secondary to DDR were observed in atherosclerotic lesions.^99^, ^106^, ^107^ Accumulated DNA damage was shown to be positively correlated with the severity of atherosclerosis in human coronary artery disease.^87^ Among different genomic insults orchestrating cardiovascular pathology, ROS represents the key endogenous culprit that leads to the formation of oxidative DNA lesions such as base oxidations, SSBs, DSBs, and telomere shortening.^5^, ^88^ An increase of BER markers following oxidative DNA damage^84^ suggests the critical role of ROS in the accumulation of DNA damage in atherosclerotic 22ques. Although mitochondrial DNA damage is also involved in the pathogenesis of CVDs,^89^ here we will only discuss the nuclear DNA damage signaling in CVDs.

DNA DSBs have been detected during genomic stress in roughly all cardiovascular cell types.^84^, ^89^, ^90^, ^91^ Theoretically, HRR is not likely to take place in postmitotic cardiomyocytes due to cell cycle restriction. Nevertheless, upregulation of BRCA1 was identified in the postischemic human myocardium.^84^ Although, in mice, depletion of BRCA1 in cardiomyocytes resulted in increased γH2AX and decreased RAD51‐foci indicating loss of DSB repair leading to DSB accumulation and impaired cardiac function in myocardial ischemia, however, there was no direct evidence of HRR repair in cardiomyocytes.^84^ Increased DNA damage and upregulation of MRN complex were observed in VSMCs of human atherosclerotic lesions, suggesting the presence of HRR.^91^ In contrast to homologous recombination, a role for NHEJ was identified in endothelial cells from atherosclerotic plaques.^92^ Here, decreased expression of lncRNA SNHG12 was attributed to decreased interaction of Ku70‐Ku80 and DNA‐PKs, leading to impaired DDR activation and vascular senescence.^92^ In an independent study, human atherosclerotic plaques were shown to contain accumulated DSBs and activated ATM in comparison to healthy tissue.^104^

DNA SSBs, on the other hand, are the most frequent outcome of oxidative stress. In proliferating cells, the most common outcome of SSBs is replication fork repression, which subsequently results in DSB formation.^94^ On the other side, cell death induced by SSBs in nondividing cells such as cardiomyocytes involves RNA polymerase and activation of PARP1 (the SSB sensor).^94^ XRCC1 depletion in cardiomyocytes increased SSBs, DDR activation, and inflammation.^95^ Moreover, the critical role of NER in cardiovascular pathology was confirmed using NER‐defective murine models, *Ercc1* ^*d/−*^ and *Xpd* ^*TTD*^, which demonstrated an enhanced vascular stiffness, senescence, and compromised vasodilator function with aging.^96^ Together, these findings highlight the significance of genomic instability and DDR activation as the central modulators of CVD progression.

### 5.2 Cancer and cardiovascular dysfunction in genomic instability syndromes (GIS)

In humans, a mutation in certain genes of DDR leads to autosomal recessive syndromes called progeroid syndromes (PS) or GIS. It should be noted that some GISs are associated with senescence, others manifest with susceptibility to cancer, and some exhibit both phenotypes.^97^ Some syndromes are important in the study of CVDs since affected patients show early onset atherosclerosis, diabetes, and dyslipidemia.^97^ Mutation of ATM in humans can lead to a distinctive GIS, called Ataxia‐telangiectasia syndrome (ATS), in homozygous patients in addition to predisposing them to cancer.^98^ ATM mutation carriers were reported to have higher mortality rates compared with noncarriers due to the occurrence of ischemic heart disease and cancer.^98^, ^99^ Heterozygous patients for the ATM allele display an enhanced risk of death from ischemic heart disease in comparison with noncarriers.^124^ As mentioned before, ATM plays a critical role in DDR by contributing to DSB repair and regulation of cell cycle checkpoint, senescence, and apoptosis. Among myriad downstream targets of ATM, p53 plays a key role by modulating the transcription of numerous target genes.^97^ However, studies investigating ATM function in *p53* knockout mice reported that there are also p53‐dependent, ATM‐independent routes leading to apoptosis and senescence.^92^, ^97^, ^100^ ATS patients demonstrate accumulated DSBs and chromosomal breakages, defective cell‐cycle checkpoints, and sensitivity to ionizing radiation. Therefore, ATM plays a critical role in maintaining genomic stability and reducing the risk of cancer and other diseases.

Werner syndrome (WS) is another GIS, which is defined by the early onset of signs of aging as well as atherosclerosis, diabetes, and a high incidence of malignancies.^108^, ^109^ The main causes of death in WS include myocardial infarction, stroke, and cancer. The WRN protein (encoded by the WRN gene) comprises a RecQ‐type helicase and an exonuclease domain and exhibits SSA activity^110^, ^111^ and telomere maintenance.^110^, ^112^ WRN‐knockdown cells are more sensitive to DSB‐inducing agents and exhibit accumulated DSBs and increased DDR rate.^113^ WRN protein contributes to HRR by interacting with BRCA1, RAD51, and RAD52. WRN also acts in concert with key molecules of NHEJ including Ku and DNA‐PKs.^97^ Extensive deletion is detected at ends joined by NHEJ in cells from WS patients.^114^ Several studies have shown that WRN‐knockdown cells exhibit defective BER and thus contain accumulated oxidative DNA damage.^115^, ^116^ Finally, WRN plays a key role in the maintenance of telomeres and telomere abrasion is the main mechanism in WS pathogenesis. Telomeres are remarkably truncated in WS cells, and telomerase introduction helps improve lifespan and decrease chromosomal aberration in these cells.^117^, ^118^, ^119^ Mice that were double‐knockout for WRN and the telomerase RNA component (TERC) manifest clinical features of WS patients,^97^, ^101^, ^102^ whereas WRN deficiency alone did not result in premature aging.^122^

In addition to ATM and WRN, accumulating evidence from recent investigations highlights the role of BRCA1, BRCA2, and p53 proteins in the pathophysiology of CVDs. Germline mutations in either one of BRCA1 and BRAC2 primarily predispose carriers to hereditary breast and ovarian cancer (HBOC) syndrome, which is inheritable in an autosomal‐dominant manner, as well as other cancer syndromes.^103^ HRR mediated by BRCA1 and BRCA2 was suggested to be a central phenomenon in the pathophysiology of CVDs as previously mentioned in this review. Studies suggest that BRCA1 and BRCA2 mutation carriers show excessive noncancerous mortality, especially at older ages,^104^, ^105^ which may be due to complications of CVDs. While p53‐induced apoptotic cell death is considered a protective route against tumor development, pro‐apoptotic p53 function is particularly damaging in the context of CVDs.^106^, ^107^ Activation of p53 by oxidative stimuli or other DNA damage sources can lead to numerous cardiovascular complications including atherosclerosis, thereby presenting a link between cancer and CVDs. Systemic deletion of BRCA1 and BRCA2 causes embryonic lethality in mice and the developing embryos exhibit defective cellular proliferation related to induction of the p53 pathway.^108^ The same study reported that heterozygous deletion of p53 can in part rescue the embryonic lethality caused by systemic BRCA1 and BRCA2 loss and that p21 mutation delays the onset of the lethality in BRCA1 mutants. These findings demonstrate that p53 and p21 are two significant modulators of cell cycle progression and play critical roles in the progression of BRCA1‐ and BRCA2‐mutant phenotypes.^108^ Intriguingly, p53 loss was reported to protect the heart from rupture and subsequent death while upregulation of p53 promoted apoptosis in cardiomyocytes.^85^, ^109^, ^110^, ^111^, ^112^ Taken together, investigations on the role of genomic instability and DDR in the prediction of cardiovascular risk can open a new window into broadening our knowledge of the common pathophysiological processes in cancer and CVDs.

### 5.3 p53: Different regulatory functions in cancer and cardiovascular diseases

The tumor suppressor p53 is the primary responder to cellular stress stimuli such as oncogene expression, DDR, ribosomal dysfunction, oxidative stress, and hypoxia. p53 activation triggers myriad cellular mechanisms which collectively induce cell cycle arrest to maintain genomic integrity, senescence, ferroptosis, or apoptosis to remove irrecoverable cells. Thereby, p53 is known as the “guardian of the genome” which prevents the accumulation of pro‐tumorigenic mutations.^112^, ^113^ TP53 is the most commonly mutated gene in human cancers.^114^ Germline mutations in TP53 result in the Li Fraumeni syndrome cancers^115^ and also are linked to adverse prognoses in numerous sporadic cancers.^114^ From a functional perspective, p53 mutants not only result in the loss of wild‐type p53 functions but can also obtain oncogenic gain‐of‐function activity, leading to a dominant negative effect through creating hetero‐tetramers with the wild‐type p53 expressed from the other nonmutated allele.^116^ Loss‐of‐function mutations in p53 render a substantial advantage during tumorigenesis by allowing cells to evade intrinsic tumor suppressive mechanisms including cell death and senescence. Gain‐of‐function mutations, on the other hand, enable p53 to acquire new transcriptional targets,^117^ such as p63, p73, NF‐Y, Sp1, ETS1/2, NF‐κB, ATM, and SMADs, modifying the metabolism, cell cycle, and apoptosis of tumor cells and promoting genomic instability, cell proliferation, metastasis, and therapy resistance.^118^

p53 also exerts essential regulatory effects on the cardiovascular system during physiological processes such as embryonic heart development and adult heart homeostasis^119^, ^120^ as well as during the development of CVDs including atherosclerosis, myocardial IRI, heart failure, diabetic‐induced or pressure‐overload maladaptive remodeling, and chemotherapy‐induced cardiotoxicity.^121^, ^122^, ^123^, ^124^ In the vasculature, p53 regulates the progression of CVDs through pro‐apoptosis, pro‐necrosis, antiangiogenesis, and pro‐autophagy activities as well as regulation of inflammation, metabolism, and cell cycle arrest.^125^, ^126^ It is important to note that p53 functions can lead to different outcomes in the cardiovascular system compared with cancer cells in terms of the pathophysiology of the disease. Contrary to the canonical role p53 plays as a tumor suppressor, its upregulation and activation can exacerbate the progression of CVDs by promoting cell death and/or phenotypical changes in the cells of the cardiovascular system. Under physiological circumstances, p53 is retained at low levels by the ubiquitin‐proteasome system.^127^ Basal levels of p53 maintain normal cardiac function and architecture by regulating the expression of cardiac architecture‐related proteins. p53 has different functions in cardiomyocytes and nonmyocytes. While p53 primarily regulates metabolism and programmed cell death in cardiomyocytes, it controls angiogenesis and cell cycle arrest in nonmyocytes. A growing body of research has shown an increased p53 expression and activation in numerous CVDs such as late‐stage heart failure.^128^ Upon stimulation by stress, p53 is subjected to several translational or posttranslational modifications such as phosphorylation, ubiquitination, acetylation, glycosylation, and SUMOylation in CVDs. p53 regulates angiogenesis by suppressing the expression of angiogenic mediators.^129^ Endothelial cell‐specific *p53* knockout was shown to promote angiogenesis in a murine hindlimb ischemia model.^130^ Endothelial p53 expression was also increased in mice fed with a high‐fat diet.^131^ Additionally, high‐fat diet‐induced eNOS (endothelial nitric oxide synthase type III enzyme) dysfunction was restored via *p53* knockdown in endothelial cells.^132^ Low‐grade expression of p53 is not pro‐apoptotic, but it results in reversible cell cycle arrest^133^ and inhibits migration of endothelial cells via downregulation of β‐3 integrin.^134^ The principal role of p53 in atherosclerosis was demonstrated using *apoE* ^*−/−*^ *p53* ^*−/−*^ double‐knockout mice fed with a high‐fat diet.^135^ These animals demonstrated increased hypercellular lesions in their aorta.^135^ Later, it was shown that p53 deficiency in subendothelial macrophages promoted atherosclerotic lesions.^136^ Furthermore, p53 plays a critical role in vascular senescence. Disturbed blood flow triggers endothelial cell apoptosis via p53 SUMOylation, a phenomenon that was ameliorated in p53^−/−^mice.^137^ In endothelial cells, prolonged IFN‐γ treatment triggered senescence through the accumulation of γH2AX foci and upregulation of p53 and p21. Additionally, ATM knockdown alleviated IFN‐γ‐induced cellular senescence.^138^ Treatment of endothelial cells with hydrogen peroxide also induced senescence through p53 and NAD‐dependent deacetylase sirtuin‐1 (SIRT1).^138^ Reduced endothelial SIRT‐1 expression during aging^139^ augments genomic instability, leading to p53 activation and exacerbation of senescence.^140^, ^141^ Taken together, these findings highlight the role of p53 activation secondary to stress stimuli such as oxidative stress and DNA damage in the modulation of endothelium function and progression of CVDs.

## 6 DNA DAMAGE RESPONSE SIGNALING IN THE PATHOPHYSIOLOGY OF CARDIOVASCULAR DISEASES

**FIGURE 4:**
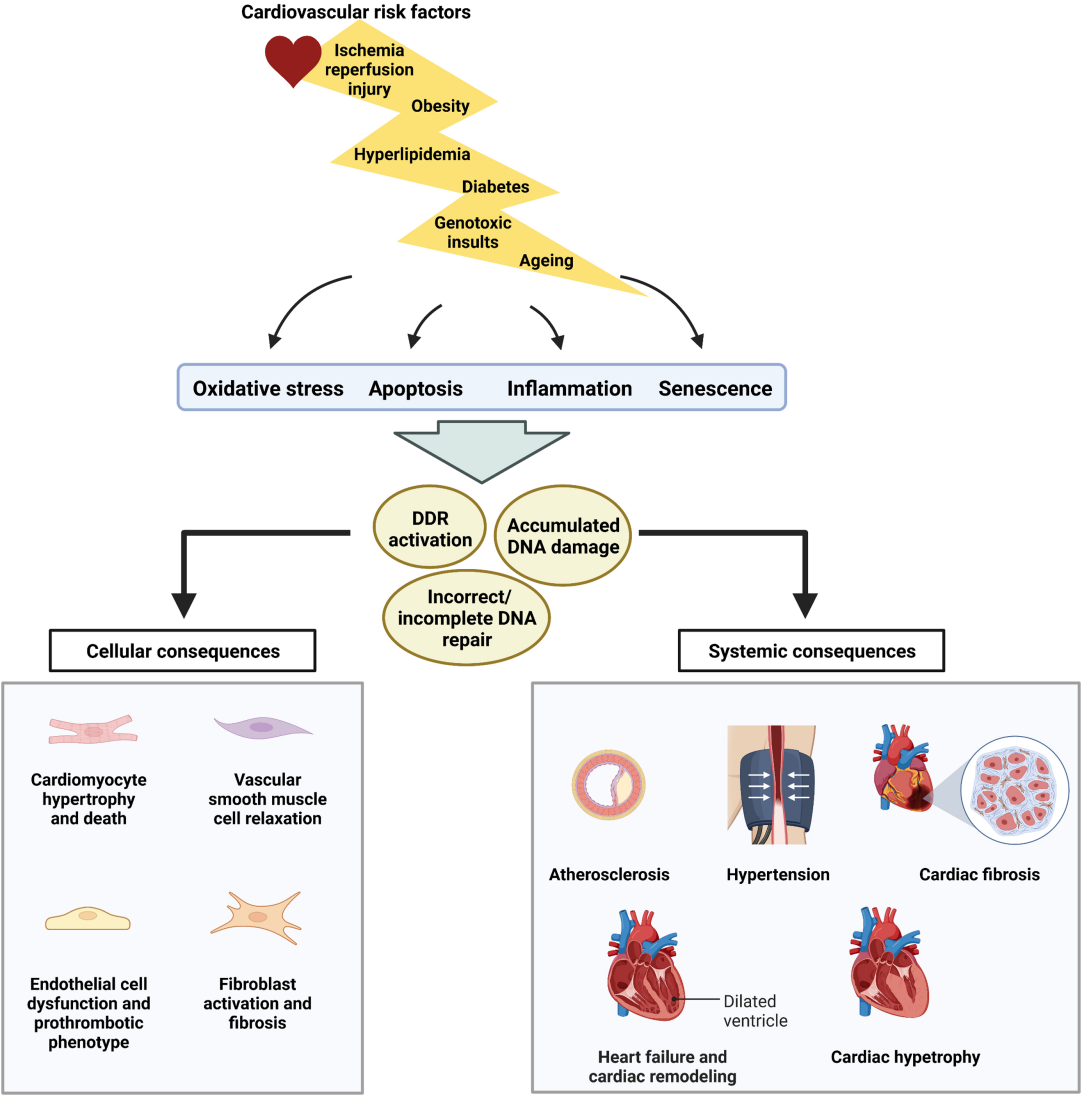
DNA damage response signaling and its cellular and systemic consequences in cardiovascular diseases. Classic cardiovascular risk factors such as aging, diabetes, hyperlipidemia, obesity, ischemia‐reperfusion injury, and genotoxic insults trigger oxidative stress, inflammation, apoptosis, and/or senescence in cells of the cardiovascular system. Together these outcomes promote DNA damage and activation of DDR mechanisms while disturbing proper DNA repair, resulting in a variety of cellular and systemic consequences in the cardiovascular system. DDR, DNA damage response.

The overall role of DDR in the pathophysiology of CVDs is briefly summarized in Figure 4.

### 6.1 Oxidative stress, atherosclerosis, and aging

It is widely accepted that factors related to the development of CVDs can trigger the generation of ROS and lead to oxidative DNA damage and subsequent senescence by targeting both nuclear and mitochondrial DNA (thoroughly reviewed in^75^, ^76^). ROS can be a by‐product of endogenous biochemical processes such as mitochondrial respiration, membrane NADH/NADPH oxidase, peroxisomes, endoplasmic reticulum, and uncoupling of NOS or exposure to exogenous stimuli such as chemotherapeutic agents (cisplatin and doxorubicin) and ionizing radiation that affect the function of the aforementioned enzymes and organelles.^142^, ^143^ ROS regulate a variety of signaling pathways that can lead to endothelial cell dysfunction, premature aging, and atherosclerosis progression.^144^, ^145^ ROS induce DNA damage by generating oxidized nucleoside bases such as 7,8‐dihydro‐8‐oxoguanine (8‐oxoG),^147^ which results in G‐A or G‐T transversions if left unrepaired. Atherogenic risk factors such as smoking and diabetes induce oxidative DNA damage, impair DDR, and accelerate the formation of mutagenic advanced glycation end products (AGEs).^148^ Increased oxidative stress and reduced antioxidant levels together with impaired DDR can gradually lead to the progression of atherosclerotic coronary disease.^148^, ^149^ Oxidized bases are typically resolved by the BER system; however, simultaneous formation of oxidized bases on opposing strands can result in the generation of DSBs by BER.^151^ Levels of 8‐oxoG and DNA strand breaks were higher in patients with atherosclerosis compared to a normal cohort with multiple reports stating a correlation in levels with disease severity.^86^, ^151^ Martinet *et al*. reported enhanced immunoreactivity to DNA damage markers and upregulation of DDR proteins in atherosclerotic plaques of carotid artery.^153^ In vitro, BRCA1‐ and BRCA2‐deficient endothelial cells are associated with increased inflammatory and oxidative stress‐induced ROS production, accumulation of DNA damage leading to apoptosis, and/or endothelial dysfunction,^153^, ^154^ which play integral roles in the development of various CVDs. In another study, human atherosclerosis was related to VSMC senescence which demonstrated elevated levels of 8‐oxoG and remarkable telomere shortening.^156^ Mice that were knockout for OGG1 (a BER protein that removes 8‐oxoG from DNA) in VSMCs showed elevated oxidative stress, DNA‐strand breaks, activation of pro‐inflammatory mechanisms, and extensive atherosclerosis.^91^ DNA damage can result in apoptosis in cells with accumulated DNA damage and impaired DDR as well as in cells that are triggered to proliferate. In atherosclerotic plaques, apoptosis is detected in endothelial cells, macrophages, and VSMCs. While VSMC apoptosis can trigger attenuation of the fibrous cap and plaque rupture, endothelial cell death is evident in both atherogenesis and plaque erosion.^157^ Apoptosis can be triggered by ROS in vascular endothelium where it is exacerbated by p53, leading to plaque rupture.^158^ Although accumulating evidence demonstrates DDR activation in atherosclerosis, cells exhibiting DNA damage are much more frequent compared with ones that exhibit apoptotic markers, indicating that DDR is activated to prevent cell death.^153^ Systemic low‐density lipoprotein receptor (LDLR) knockout mice that were fed a high‐lipid diet expressed higher p53 levels along with disturbed flow‐induced senescence mediated by the p53–p21 pathway.^159^ p21 knockout mice exhibited accelerated atherosclerosis following high‐lipid diet consumption compared with their wild‐type littermates.^160^ DDR markers in human plaques are related to activation of BER or nonspecific repair pathways.^153^ Mercer *et al*. showed that ATM knockout in *ApoE*‐null mice leads to accelerated atherosclerosis.^161^ In plaques of thoracic aorta derived from cholesterol‐fed rabbits, DNA damage was associated with the upregulation of DDR enzymes such as PARP1 and XRCC1.^162^ Although DNA breaks were normalized following four weeks of dietary lipid reduction, a remarkable reduction of 8‐oxoG was only evident after a long period of diet modification, indicating the prolonged existence of DNA damage in atherosclerosis. Moreover, DDR proteins were significantly downregulated when switched to a normal diet.^162^ Taken together, these studies show that DNA damage and the subsequent DDR activation play central roles in atherosclerosis and may be repairable, at least in the early stages of atherosclerosis.

A great amount of ROS is produced throughout vascular aging.^163^ This ROS can be either a product of eNOS, when it is in the uncoupled state,^164^ or of other resources such as NADPH oxidase or mitochondria, which form free radicals upon interacting with NO. Overproduction of ROS can ultimately culminate in decreased NO bioavailability, formation of SSBs, 8‐oxoG, and other oxidative lesions in vascular endothelial cells.^165^ Aberrant eNOS activity is closely related to genomic instability and dysfunctional vascular endothelial cells as observed in diseased and aged blood vessels. Downregulation of eNOS mRNA was correlated with overt DNA damage in human endothelial cells treated with oxidized LDL *in vitr*o^155^ and with advanced atherosclerotic plaques *in vivo*.^153^ eNOS knockout mice demonstrated a variety of cardiovascular complications such as altered vascular remodeling, systemic hypertension, abnormal angiogenesis, and a prothrombotic phenotype.^165^, ^166^, ^167^, ^168^, ^169^ Apart from the critical role, eNOS plays in the initiation of DNA damage, genomic instability itself can result in the disturbance of NO signaling. Mice with genetic instability due to deficient ERCC1 (*Ercc1* ^*d/−*^ mice; involved in the NER pathway) demonstrated early onset vascular cell senescence, vasodilatory dysfunction, elevated blood pressure, and enhanced vascular stiffness. The impaired vasodilatory function was correlated with increased eNOS‐activating phosphorylation at serine 1177 residue which subsequently resulted in reduced activation and production of eNOS and decreased NO‐mediated vasodilation.^95^ Uncoupling of eNOS due to decreased tetrahydrobiopterin (BH4) bioavailability can lead to a switch from NO to ROS production. Similar to Ercc1^d/−^ mice, age‐dependent endothelial cell‐mediated vasodilation was impaired in mice with XPD deficiency (*Xpd* ^*TTD*^ mice; another gene involved in the NER mechanism).^95^ Moreover, mice that were transgenic for APE1/ref‐1 (apurinic/apyrimidinic endonuclease 1/redox factor 1; plays major roles in DDR) manifested decreased vascular NO levels, dysfunctional endothelial cell‐dependent vascular tone, and systemic hypertension.^99^ Taken together, aberrant eNOS activation and genomic instability can ultimately lead to progressive endothelial cell dysfunction which promotes vascular aging.

### 6.2 Metabolic stress

#### 6.2.1 Diabetes

Patients with type 1 or type 2 diabetes show enhanced serum levels of glucose and AGEs which are considered diagnostic markers. As a result of obesity‐related insulin resistance, type 2 diabetic patients exhibit increased levels of serum insulin and free fatty acids during the early stages of the disease. These pathophysiological factors were reported to induce DNA damage *in vitro* which may explain why diabetic patients show enhanced DNA damage *in vivo*.^171^ Hyperglycemia was shown to promote DNA DSBs in endothelial cells as well as increase 8‐oxoG levels in endothelial and tubular cells.^171^, ^172^ The genotoxicity of AGEs was proven using numerous cell lines such as pig kidney cells,^174^ human liver and colon cells,^175^ and mouse podocytes.^176^ AGEs were also able to cause oxidation of DNA bases and trigger the generation of 8‐oxoG in VSMCs.^177^ In diabetes‐associated mutation, DNA damage caused by ROS can take place either through a direct or indirect pathway. Increased plasma levels of glucose, AGEs, insulin, and free fatty acids—all initiate ROS generation leading to direct DNA damage in type 2 diabetes.^178^ Additionally, diabetic patients demonstrate reduced antioxidative capacity such as decreased glutathione synthesis, which can result in susceptibility to oxidative damage.^179^ Indirect diabetes‐associated DNA damage can take place *via* various signaling pathways such as the PI3K‐Akt‐tuberin pathway.^180^ Blasiak *et al*. reported decreased DDR efficacy in type 2 diabetic patients.^181^ Since impaired DDR may increase the proneness of cancer development,^181^ diabetic patients may also develop a greater risk for carcinogenesis.^182^ The effect of hyperglycemia on cellular oxidative stress and 8‐oxoG levels has been investigated in human microvascular^183^ and human umbilical vein endothelial cells.^184^

#### 6.2.2 Hyperlipidemia

Studies have demonstrated that obese patients who show enhanced risk for cancer also showed higher serum levels of free fatty acids and 8‐oxoG, strengthening the notion that there is a possible correlation among free fatty acids, DNA damage, and carcinogenesis.^185^ Atheroma obtained from *ApoE*‐null mice fed a high‐fat diet contained increased DNA DSBs along with enhanced levels of poly ADP ribose, arising from PARP activity.^161^, ^185^ In addition, enhanced DNA damage, poly ADP ribose, and cell death were clearly detected within the atheromatous plaques in this model.^186^ Godschalk *et al*.^187^ assessed DNA etheno adducts in aortic cells derived from *ApoE*‐null mice fed either a low‐ or a high‐lipid diet and reported that the number of exocyclic adducts was inversely associated with the levels of total serum cholesterol. However, the expression of APE1/Ref1 was enhanced in these mice, suggesting that increased DDR caused by genotoxic lipid oxidation products is probably responsible for the observed decrease in DNA damage.^187^ Etheno adducts were also found in human atherosclerotic plaques as reported by the same researchers. Although few studies have confirmed the casual correlation between hyperlipidemia and DNA damage,^160^, ^187^, ^188^, ^189^, ^190^ it appears that consuming a high‐lipid diet can induce DNA damage in the cardiovascular system.

#### 6.2.3 Obesity

Obesity is involved in the early onset of diabetes and atherosclerosis‐associated diseases and it enhances the risk of death from CVDs.^192^ Mutations of DDR genes in mice and humans lead to a phenotype that is similar to obesity‐related metabolic and cardiovascular dysfunctions.^193^ A higher frequency of DNA lesions including SSB, DSB, and oxidized bases was observed in lymphocytes derived from obese patients.^193^, ^194^, ^195^ It is now well accepted that chronic energy overload in obesity leads to elevated ROS generation and creates a chronic inflammatory condition.^197^ Production of pro‐inflammatory adipokines as a result of excessive fat accumulation in adipocytes induces infiltration of T cells and macrophages.^198^ Accumulation of immune cells in adipose tissue triggers the production of ROS by NOX2, the NADPH oxidase produced in inflammatory cells. Smooth muscle cells and adipocytes exposed to excessive free fatty acids or hyperglycemia displayed enhanced mitochondrial ROS generation.^198^, ^199^, ^200^ Obesity‐associated chronic inflammation was strongly correlated with the instigation of DNA damage.^197^ Activated macrophages release cytokines such as IL‐6 and TNF‐α which are capable of inducing DNA damage in nontargeting tissues remote from the inflammation site.^201^, ^202^ Translocation of cytokines to distinct tissues of the body triggers resident macrophages to release pro‐inflammatory mediators including NO, NOS, COX2, superoxide, and ROS,^203^, ^204^ which all culminate in oxidative DNA damage. In young females, body mass index was inversely associated with NER capacity.^206^ Obesity was also reported to dysregulate DSB repair triggered by genotoxic factors.^207^ Studies have reported inhibition of DDR enzymes associated with oxidative stress in obesity.^207^, ^208^ Barouch *et al*. demonstrated that cardiomyocyte apoptosis was linked to enhanced DNA damage and reduced survival rate in obese mice.^210^

### 6.3 Myocardial ischemia‐reperfusion injury (IRI)

Myocardial IRI is the most common consequence of ischemic heart disease which results in apoptosis and necrosis along with the transient decrease of contractility in surviving myocardial tissue.^212^ Shortly after total or partial coronary artery obstruction, ischemic myocardium utilizes anaerobic glycolysis as the main mechanism for generating ATP, leading to excessive production of intracellular H^+.^ ^211^, ^212^ When reperfusion and reoxygenation take place, the heart tissue will operate at a relatively inefficient rate due to the imbalance of electrolytes^214^ and the resultant myocardial dysfunction, microvascular injury, and arrhythmias.^214^, ^215^, ^216^ The most notable result of reperfusion is an ROS burst which is a main contributory factor to IRI. The ROS burst together with a variety of IRI‐induced pathophysiological events that arise from intense mitochondrial redox conditions that exacerbate ROS generation. Overproduction of ROS activates oxidative DNA damage signaling and PARP1, leading to decreased intracellular ATP and NAD^+.^ ^217^, ^218^ NAD^+^ is a cosubstrate for sirtuin family proteins (SIRT1‐7), all of which play a critical role in regulating redox homeostasis and are associated with CVDs.^219^, ^220^ Intracellular depletion of NAD^+^ attenuates the activity of SIRT1^222^ and SIRT3,^223^ further disturbing mitochondrial biogenesis and antioxidant defense, which leads to mitochondrial dysfunction, one of the hallmarks of IRI.^224^ Numerous investigations have confirmed the correlation between myocardial IRI and ROS‐induced oxidative DNA damage.^225^ The production of 8‐oxoG was directly related to the IRI severity in a study on rat hearts.^226^ In another study on rat myocardium, the level of 8‐oxoG was gradually enhanced as a function of reperfusion time and was totally inhibited by means of an ROS scavenger.^227^ These findings validate the remarkable role of oxidative DNA damage in the development of myocardial IRI. Dysfunctional DDR proteins were also reported to negatively impact cell proliferation, apoptosis, and mitochondrial function, enhancing the risk of atherosclerosis, metabolic syndromes, and ischemic heart disease.^161^, ^227^ Collectively, although interventional strategies are essential for protecting the heart from ischemic injury, they can be inevitably entangled by IRI. Therefore, the lack of sufficient clinical data and inadequate pharmacodynamic and pharmacokinetic studies prompt a critical demand for efficient treatments for IRI in ischemic heart disease.

### 6.4 Heart failure and cardiac remodeling

Recent studies demonstrated the role of DNA damage in the setting of heart failure in rodent models^95^ and humans.^231^ In mice, cardiomyocyte‐specific loss of BRCA1 or BRCA2 is shown to exacerbate myocardial infarction‐ and doxorubicin‐induced accumulation of DNA damage, respectively, eventually leading to heart failure.^85^, ^231^ Studies have reported activation of DNA glycosylase toward 3‐methyladenine and uracil in the infarcted and noninfarcted areas of the left ventricle in a rodent model of heart failure.^233^ Given the central role of DNA glycosylases in BER, these findings demonstrate an increase in deaminated and alkylated base lesions in heart failure. PARP1 deficiency in a murine model of aortic banding decreased translocation of apoptosis‐inducing factors and myocardial hypertrophy.^234^ Studies have exhibited a role for BRCA1 and BRCA1‐associated protein 2 (BRAP2) in an experimental model of right ventricular hypertrophy.^235^ Another study demonstrated that mechanical loading induced cardiac hypertrophy associated with overexpression of BRCA1.^236^ In another study, single nucleotide polymorphisms in BRAP2 were significantly associated with susceptibility to myocardial infarction.^237^ Higo *et al*. reported that DDR activation secondary to DNA SSBs increases the expression of pro‐inflammatory cytokines via the NF‐κB pathway in cardiomyocytes of the pressure overload‐induced failing heart. Additionally, they showed that XRCC1 knockout mice had more severe heart failure, a phenomenon that was later restored by ATM deletion.^95^ In another study, *Cao et al*. identified a causal role for Ascc2 (activating signal cointegrator 1 complex subunit 2; an enzyme involved in DNA alkylation damage repair) in heart failure with preserved ejection fraction.^238^

## 7 TOWARD INTERVENTIONAL STRATEGIES IN CARDIOVASCULAR DYSFUNCTION CAUSED BY GENOMIC INSTABILITY

Given the significant contribution of DNA damage in the pathophysiology of CVDs, therapeutic strategies targeted toward genomic instability and DDR are proposed to lower CVDs‐related morbidity and mortality. In general, such strategies can either inhibit the initiation of DNA damage or inactivate the DDR mechanisms in various disease stages (Figure 1).

### 7.1 Preventing DNA damage in cardiovascular diseases

#### 7.1.1 Natural antioxidants

Preclinical and clinical studies have demonstrated that polyphenols such as resveratrol alleviate DNA damage in CVDs^239^ via activating antioxidant enzymes and scavenging ROS.^240^ Although therapeutically effective in preclinical investigations,^241^ randomized controlled trials of supplementary antioxidants such as vitamins C and E have not shown remarkable cardiovascular benefits in humans.^241^, ^242^ The inconsistency among these studies could be due to inadequate methodological design of trials, diverse baseline status of antioxidants, and lack of reliable oxidative stress biomarkers for clinical trials.

#### 7.1.2 Statins

Statins exert their pharmacological effects via alleviation of DNA damage in CVDs such as atherosclerosis,^243^, ^244^ dyslipidemia,^245^, ^246^ myocardial infarction,^248^ and chemotherapy‐induced cardiopathy.^249^ Statins decrease oxidative DNA damage and suppress DDR signaling. Statins can trigger phosphorylation of the ubiquitin ligase MDM2, leading to p53 degradation. Atorvastatin therapy inhibited ATM and ATR and decreased VSMC senescence and apoptosis in atherosclerosis.^244^ Statins can facilitate DDR through phosphorylation of human double minute protein Hdm2, resulting in Hdm2 dissociation from NBS‐1 and inhibition of NBS‐1 degradation. Additionally, statin therapy abrogates ionizing radiation‐induced DDR including p53 and CHK1 activation in human endothelial cells.^250^

#### 7.1.3 Angiotensin‐converting enzyme inhibitors (ACEIs)/angiotensin II receptor blockers (ARBs)

Elevated levels of angiotensin II cause oxidative stress in endothelial dysfunction and atherosclerosis.^250^ Ang II triggers oxidative DNA damage and accelerates vascular senescence via AT1 receptor in humans.^251^ ACEIs/ARBs exert cardiovascular protection by attenuating oxidative DNA damage.^251^, ^252^ The ARB losartan can decrease oxidative DNA damage and cellular senescence via inhibiting telomere‐dependent replicative senescence, or telomere‐independent stress‐induced premature senescence.^251^ Oxidative DNA damage and myocardial infarction severity were decreased by valsartan and ramipril treatment in an experimental myocardial infarction model.^254^ Moreover, the administration of ARBs prevented oxidative DNA damage in hypertensive mice.^255^

#### 7.1.4 β‐blockers

Although little is known regarding the mechanism of β‐adrenergic receptor blockade on DNA damage, experimental studies have shown that β‐blockers exert cardiovascular protective effects by inhibiting DNA damage.^256^, ^257^, ^258^ Early administration of carvedilol‐alleviated doxorubicin‐induced DNA damage and cardiotoxicity.^258^ Carvedilol therapy also reduced levels of 8‐oxoG and ameliorated heart failure in patients with dilated cardiomyopathy^257^ and reduced oxidative DNA damage in patients with hypertension.^259^

### 7.2 DNA damage response blockade: novel targets for the treatment of cardiovascular diseases

#### 7.2.1 PARP inhibition

Accumulating evidence confirmed that PARP enzymes are aberrantly activated in CVDs,^188^, ^259^ and PARP inhibition is rendered therapeutically effective in preclinical and clinical investigations of CVDs.^260^, ^261^ INO‐1001 reduced infarct size and improved cardiac function in myocardial IRI^233^, ^262^ and became the first PARP inhibitor to enter clinical trials.^264^ In this trial, PARP inhibition reduced postmyocardial infarction‐associated inflammation via lowering plasma C‐reactive protein and IL‐6 levels. Pharmaceutical inhibition of PARP1 activity in mice with type 2 diabetes could remarkably improve vascular tone and function through NF‐κB pathway regulation.^265^ In another independent study, INO‐1001 inhibited pressure overload‐induced reduction in cardiac contractility and prevented cardiac fibrosis, hypertrophy, and apoptosis in a murine model of heart failure.^234^

#### 7.2.2 ATM inhibition

Genetic ablation of ATM and pharmacological inactivation by KU60019 improved cardiac function secondary to pressure overload.^266^ The lncRNA Caren preserved cardiac function during pressure overload by inhibition of ATM and activation of mitochondrial bioenergetics.^91^ In Caren loss‐of‐function mice, KU60019 could relatively rescue cardiac function. Although ATM inhibition has shown promising cardioprotective effects in heart failure, several studies have reported aberrant ATM activation in myocardial infarction.^266^, ^267^, ^268^, ^269^ ATM deficiency attenuated postmyocardial infarction cardiac dysfunction, dilation, fibrosis, and increased apoptosis.^267^ Furthermore, ATM was shown to trigger cardiac inflammation during myocardial infarction.^268^ In ATM heterozygous knockout mice, cardiac remodeling was attenuated after myocardial infarction. KU60019 was recently shown to reduce infarct size and attenuate systolic dysfunction in myocardial infarction.^270^ While these studies hold promise of ATM inhibition in myocardial infarction, several investigations demonstrated that ATM deficiency aggravated postmyocardial infarction cardiac dysfunction and remodeling via disturbed autophagy and angiogenesis.^270^, ^271^ Based on these studies, ATM inhibition diminished cardiac inflammation, remodeling, and dysfunction 1–7 days postmyocardial infarction, whereas aggravating cardiomyocyte apoptosis, fibrosis, and cardiac hypertrophy was 14–28 days postmyocardial infarction.

#### 7.2.3 ATR inhibition

Excessive ATR‐p53 activation secondary to replication stress‐induced DNA damage was reported in a model of Mybpc3^−/−^ cardiomyopathy.^273^ Here, AZD6738 (a highly selective ATR inhibitor) was able to decrease cardiac remodeling and p53 depletion in cardiomyocytes imitated the cardioprotective function of AZD6738.

#### 7.2.4 DNA‐PK inhibition

DNA‐PKs are aberrantly activated in various CVDs such as myocardial IRI,^274^ cardiac hypertrophy,^266^ atherosclerosis,^93^, ^155^, ^274^ and diabetic cardiomyopathy.^276^ Genetic and pharmacological inhibition of DNA‐PKs showed favorable effects in atherosclerosis. The selective DNA‐PK inhibitors DMNB and NU7024 could prevent NOR‐1‐dependent proliferation of aortic VSMCs in humans.^275^ Moreover, DNA‐PK inhibition was shown to decrease neointimal lesion size following wire injury. DNA‐PK was excessively activated in response to IRI‐induced stress, and DNA‐PK inhibition was reported to have protective effects.^274^ Interestingly, cardiomyocyte‐specific DNA‐PK depletion prevented inflammation and apoptosis and preserved cardiac function during IRI injury.

#### 7.2.5 CHK1/2 inhibition

Several investigations have recently reported a role for CHK1/2 kinases in pulmonary artery hypertension^274^ and heart failure.^275^ Cardiomyocyte apoptosis is associated with CHK2 activation and telomere shortening.^275^ CHK1 was shown to be upregulated in distal pulmonary arteries and VSMCs of patients with pulmonary arterial hypertension.^273^, ^275^ CHK1 promoted VSMC resistance to apoptosis and proliferation. The CHK1 inhibitor MK8776, previously tested for clinical efficacy, improved hemodynamic parameters, and attenuated vascular remodeling in an experimental model of pulmonary artery hypertension,^274^ suggesting a therapeutic potential for CHK1 inhibitors in the management of pulmonary artery hypertension.

#### 7.2.6 p53 inhibition

Several upstream kinases such as ATM, ATR, DNA‐PK, CHK1, and CHK2^280^ regulate the phosphorylation and activation of p53. p53 activation was reported in a variety of CVDs such as cardiac remodeling, heart failure, diabetic cardiomyopathy, myocardial IRI, and chemotherapy‐induced cardiotoxicity. Moreover, p53 plays a cardinal role in the pathogenesis of CVDs by mediating autophagy, apoptosis, angiogenesis, necrosis, senescence, and metabolic alterations. Genetic and pharmacological loss‐of‐function of p53 demonstrates beneficial effects on cardiovascular health.^280^ The therapeutic potential of p53 in CVDs is demonstrated in the mouse model, where deletion of one p53 allele is sufficient to protect the heart in cardiomyocyte‐specific BRCA1 knockout mice.^85^ The p53 inhibitor pifithrin is currently utilized in preclinical models of CVDs to further assess its therapeutic efficacy.

## 8 FUTURE PERSPECTIVE

Inherited cancer syndrome is characterized by an increased risk of certain types of cancer that accounts for approximately 10% of all cancer cases.^280^ Inherited cancer syndromes are caused by heritable mutations in specific genes that lead to specific patterns of cancer such as early onset tumor development or developing more than one type of cancer in the same person.^280^, ^281^ Mutations of BRCA1 and/or BRCA2 result in HBOC that enhances the chance of developing breast, ovarian, prostate, pancreatic, and colon cancers.^283^ BRCA genes are inheritable in an autosomal‐dominant manner and tend to be very penetrant. BRCA‐associated tumors exhibit distinctive manifestations, clinical features, and pathologic profiles including younger patient age, advanced tumors, and aggressive nature. Current standard methods for experimental assessment of BRCA genes include comprehensive sequencing and examination of broad genomic rearrangements. Thorough knowledge of the molecular pathways involved in the initiation and progression of these malignancies will open a window on the behavior of these tumors and differential diagnosis of affected patients, supporting healthcare providers to make appropriate decisions regarding therapies. Recent investigations have revealed surprising similarities of molecular pathways involved in cancer and CVDs. Cumulative data suggest that DDR is activated in a variety of CVDs, indicating a close relation between CVDs and DNA damage or deficient DDR. Studies suggest that BRCA1 and BRCA2 mutation carriers show increased nonneoplastic mortality, especially at older ages.^103^, ^104^ While the causal factors of nonneoplastic death in these patients are still unidentified, the data presented in this review raise the likelihood that excess mortality can be mediated by enhanced rates of cardiovascular complications. From a pharmacological point of view, comprehensive knowledge of the DDR network will provide opportunities for the development of novel personalized therapeutics based on specific disease profiles. Furthermore, the knowledge that DDR is involved in both cancer and CVDs offers new opportunities to regulate oxidative DNA damage and target DDR pathways for the rational development of novel treatments.

## AUTHOR CONTRIBUTIONS

KS and SN conceived and SN wrote the first draft of this review. SN made the figures. KS and SN revised and finalized the review.

## FUNDING INFORMATION

This work was supported by a grant from the Canadian Institutes of Health Research to KS (grant/award no. FRN # 153216).

## CONFLICT OF INTEREST

None.

## ETHICS STATEMENT

Not applied.

## CONSENT STATEMENT

Not applied.

## Data Availability

Data sharing is not applicable to this article as no new data were created or analyzed in this study.

## References

[1] Lindahl T , Barnes DE . Repair of endogenous DNA damage. Cold Spring Harb Symp Quant Biol. 2000;65:127‐133. 12760027 10.1101/sqb.2000.65.127

[2] Caldecott KW . Mammalian single‐strand break repair: mechanisms and links with chromatin. DNA Repair. 2007;6(4):443‐453. 17118715 10.1016/j.dnarep.2006.10.006

[3] Ghosal G , Chen J . DNA damage tolerance: a double‐edged sword guarding the genome. Transl Cancer Res. 2013;2(3):107‐129. 24058901 10.3978/j.issn.2218-676X.2013.04.01 PMC3779140

[4] Yang J , Yu Y , Hamrick HE , Duerksen‐Hughes PJ . ATM, ATR and DNA‐PK: initiators of the cellular genotoxic stress responses. Carcinogenesis. 2003;24(10):1571‐1580. 12919958 10.1093/carcin/bgg137

[5] Hoeijmakers JHJ . DNA damage, aging, and cancer. N Engl J Med. 2009;361(15):1475‐1485. 19812404 10.1056/NEJMra0804615

[6] Jackson SP , Bartek J . The DNA‐damage response in human biology and disease. Nature. 2009;461(7267):1071‐1078. 19847258 10.1038/nature08467 PMC2906700

[7] Ciccia A , Elledge SJ . The DNA damage response: making it safe to play with knives. Mol Cell. 2010;40(2):179‐204. 20965415 10.1016/j.molcel.2010.09.019 PMC2988877

[8] Khanna KK , Jackson SP . DNA double‐strand breaks: signaling, repair and the cancer connection. Nat Genet. 2001;27(3):247‐254. 11242102 10.1038/85798

[9] Kusakabe M , Onishi Y , Tada H , et al. Mechanism and regulation of DNA damage recognition in nucleotide excision repair. Genes and Environment. 2019;41(1):2. 30700997 10.1186/s41021-019-0119-6 PMC6346561

[10] Shah P , He YY . Molecular regulation of UV‐induced DNA repair. Photochem Photobiol. 2015;91(2):254‐264. 25534312 10.1111/php.12406 PMC4355264

[11] Schärer OD . Nucleotide excision repair in eukaryotes. Cold Spring Harb Perspect Biol. 2013;5(10):a012609. 24086042 10.1101/cshperspect.a012609 PMC3783044

[12] Cleaver JE , Lam ET , Revet I . Disorders of nucleotide excision repair: the genetic and molecular basis of heterogeneity. Nat Rev Genet. 2009;10(11):756‐768. 19809470 10.1038/nrg2663

[13] Zharkov DO . Base excision DNA repair. Cell Mol Life Sci. 2008;65(10):1544‐1565. 18259689 10.1007/s00018-008-7543-2 PMC11131669

[14] Krokan HE , Bjørås M . Base excision repair. Cold Spring Harb Perspect Biol. 2013;5(4):a012583. 23545420 10.1101/cshperspect.a012583 PMC3683898

[15] Luo M , He H , Kelley MR , Georgiadis MM . Redox regulation of DNA repair: implications for human health and cancer therapeutic development. Antioxid Redox Signal. 2010;12(11):1247‐1269. 19764832 10.1089/ars.2009.2698 PMC2864659

[16] Martin SA , Lord CJ , Ashworth A . Therapeutic targeting of the DNA mismatch repair pathway. Clin Cancer Res. 2010;16(21):5107‐5113. 20823149 10.1158/1078-0432.CCR-10-0821

[17] Jiricny J . The multifaceted mismatch‐repair system. Nat Rev Mol Cell Biol. 2006;7(5):335‐346. 16612326 10.1038/nrm1907

[18] Shaheen M , Allen C , Nickoloff JA , Hromas R . Synthetic lethality: exploiting the addiction of cancer to DNA repair. Blood. 2011;117(23):6074‐6082. 21441464 10.1182/blood-2011-01-313734

[19] Diaz‐Padilla I , Romero N , Amir E , et al. Mismatch repair status and clinical outcome in endometrial cancer: a systematic review and meta‐analysis. Crit Rev Oncol Hematol. 2013;88(1):154‐167. 23562498 10.1016/j.critrevonc.2013.03.002

[20] Li YH , Wang X , Pan Y , Lee DH , Chowdhury D , Kimmelman AC . Inhibition of non‐homologous end joining repair impairs pancreatic cancer growth and enhances radiation response. PLoS One. 2012;7(6):e39588. 22724027 10.1371/journal.pone.0039588 PMC3377637

[21] Dueva R , Iliakis G . Alternative pathways of non‐homologous end joining (NHEJ) in genomic instability and cancer. Transl Cancer Res. 2013;2(3):163‐177.

[22] Scott SP , Pandita TK . The cellular control of DNA double‐strand breaks. J Cell Biochem. 2006;99(6):1463‐1475. 16927314 10.1002/jcb.21067 PMC3088996

[23] Mills KD , Ferguson DO , Alt FW . The role of DNA breaks in genomic instability and tumorigenesis. Immunol Rev. 2003;194:77‐95. 12846809 10.1034/j.1600-065x.2003.00060.x

[24] Helleday T . Homologous recombination in cancer development, treatment and development of drug resistance. Carcinogenesis. 2010;31(6):955‐960. 20351092 10.1093/carcin/bgq064

[25] Symington LS , Gautier J . Double‐strand break end resection and repair pathway choice. Annu Rev Genet. 2011;45:247‐271. 21910633 10.1146/annurev-genet-110410-132435

[26] Lee JH , Paull TT . Direct activation of the ATM protein kinase by the Mre11/Rad50/Nbs1 complex. Science. 2004;304(5667):93‐96. 15064416 10.1126/science.1091496

[27] Lee JH , Paull TT . ATM activation by DNA double‐strand breaks through the Mre11‐Rad50‐Nbs1 complex. Science. 2005;308(5721):551‐554. 15790808 10.1126/science.1108297

[28] San Filippo J , Sung P , Klein H . Mechanism of eukaryotic homologous recombination. Annu Rev Biochem. 2008;77:229‐257. 18275380 10.1146/annurev.biochem.77.061306.125255

[29] Yang H , Li Q , Fan J , Holloman WK , Pavletich NP . The BRCA2 homologue Brh2 nucleates RAD51 filament formation at a dsDNA‐ssDNA junction. Nature. 2005;433(7026):653‐657. 15703751 10.1038/nature03234

[30] Jensen RB , Carreira A , Kowalczykowski SC . Purified human BRCA2 stimulates RAD51‐mediated recombination. Nature. 2010;467(7316):678‐683. 20729832 10.1038/nature09399 PMC2952063

[31] Thorslund T , McIlwraith MJ , Compton SA , et al. The breast cancer tumor suppressor BRCA2 promotes the specific targeting of RAD51 to single‐stranded DNA. Nat Struct Mol Biol. 2010;17(10):1263‐1265. 20729858 10.1038/nsmb.1905 PMC4041013

[32] Prakash R , Zhang Y , Feng W , Jasin M . Homologous recombination and human health: the roles of BRCA1, BRCA2, and associated proteins. Cold Spring Harb Perspect Biol. 2015;7(4):a016600. 25833843 10.1101/cshperspect.a016600 PMC4382744

[33] Graham TGW , Walter JC , Loparo JJ . Two‐stage synapsis of DNA ends during non‐homologous end joining. Mol Cell. 2016;61(6):850‐858. 26990988 10.1016/j.molcel.2016.02.010 PMC4799494

[34] Blackford AN , Jackson SP . ATM, ATR, and DNA‐PK: the trinity at the heart of the DNA damage response. Mol Cell. 2017;66(6):801‐817. 28622525 10.1016/j.molcel.2017.05.015

[35] Gottlieb TM , Jackson SP . The DNA‐dependent protein kinase: requirement for DNA ends and association with Ku antigen. Cell. 1993;72(1):131‐142. 8422676 10.1016/0092-8674(93)90057-w

[36] Nick McElhinny SA , Snowden CM , McCarville J , Ramsden DA . Ku recruits the XRCC4‐ligase IV complex to DNA ends. Mol Cell Biol. 2000;20(9):2996‐3003. 10757784 10.1128/mcb.20.9.2996-3003.2000 PMC85565

[37] Ahnesorg P , Smith P , Jackson SP . XLF interacts with the XRCC4‐DNA ligase IV complex to promote DNA nonhomologous end‐joining. Cell. 2006;124(2):301‐313. 16439205 10.1016/j.cell.2005.12.031

[38] Buck D , Malivert L , de Chasseval R , et al. Cernunnos, a novel nonhomologous end‐joining factor, is mutated in human immunodeficiency with microcephaly. Cell. 2006;124(2):287‐299. 16439204 10.1016/j.cell.2005.12.030

[39] Ochi T , Blackford AN , Coates J , et al. DNA repair. PAXX, a paralog of XRCC4 and XLF, interacts with Ku to promote DNA double‐strand break repair. Science. 2015;347(6218):185‐188. 25574025 10.1126/science.1261971 PMC4338599

[40] Chakraborty A , Tapryal N , Venkova T , et al. Classical non‐homologous end‐joining pathway utilizes nascent RNA for error‐free double‐strand break repair of transcribed genes. Nat Commun. 2016;5(7):13049. 10.1038/ncomms13049 PMC5059474 27703167

[41] Davis AJ , Chen DJ . DNA double strand break repair via non‐homologous end‐joining. Transl Cancer Res. 2013;2(3):130‐143. 24000320 10.3978/j.issn.2218-676X.2013.04.02 PMC3758668

[42] Bunting SF , Nussenzweig A . End‐joining, translocations and cancer. Nat Rev Cancer. 2013;13(7):443‐454. 23760025 10.1038/nrc3537 PMC5724777

[43] Zha S , Guo C , Boboila C , et al. ATM damage response and XLF repair factor are functionally redundant in joining DNA breaks. Nature. 2011;469(7329):250‐254. 21160472 10.1038/nature09604 PMC3058373

[44] Iliakis G , Murmann T , Soni A . Alternative end‐joining repair pathways are the ultimate backup for abrogated classical non‐homologous end‐joining and homologous recombination repair: implications for the formation of chromosome translocations. Mutat Res Genet Toxicol Environ Mutagen. 2015;793:166‐175. 26520387 10.1016/j.mrgentox.2015.07.001

[45] Yu AM , McVey M . Synthesis‐dependent microhomology‐mediated end joining accounts for multiple types of repair junctions. Nucleic Acids Res. 2010;38(17):5706‐5717. 20460465 10.1093/nar/gkq379 PMC2943611

[46] Simsek D , Jasin M . Alternative end‐joining is suppressed by the canonical NHEJ component Xrcc4‐ligase IV during chromosomal translocation formation. Nat Struct Mol Biol. 2010;17(4):410‐416. 20208544 10.1038/nsmb.1773 PMC3893185

[47] Deriano L , Roth DB . Modernizing the nonhomologous end‐joining repertoire: alternative and classical NHEJ share the stage. Annu Rev Genet. 2013;47:433‐455. 24050180 10.1146/annurev-genet-110711-155540

[48] Hartlerode AJ , Scully R . Mechanisms of double‐strand break repair in somatic mammalian cells. Biochem J. 2009;423(2):157‐168. 19772495 10.1042/BJ20090942 PMC2983087

[49] Bhargava R , Onyango DO , Stark JM . Regulation of single‐strand annealing and its role in genome maintenance. Trends Genet. 2016;32(9):566‐575. 27450436 10.1016/j.tig.2016.06.007 PMC4992407

[50] Deans AJ , West SC . DNA interstrand crosslink repair and cancer. Nat Rev Cancer. 2011;11(7):467‐480. 21701511 10.1038/nrc3088 PMC3560328

[51] Garaycoechea JI , Crossan GP , Langevin F , Daly M , Arends MJ , Patel KJ . Genotoxic consequences of endogenous aldehydes on mouse haematopoietic stem cell function. Nature. 2012;489(7417):571‐575. 22922648 10.1038/nature11368

[52] Hashimoto S , Anai H , Hanada K . Mechanisms of interstrand DNA crosslink repair and human disorders. Genes Environ. 2016;38:9. 27350828 10.1186/s41021-016-0037-9 PMC4918140

[53] Wang Y , Leung JW , Jiang Y , et al. FANCM and FAAP24 maintain genome stability via cooperative as well as unique functions. Mol Cell. 2013;49(5):997‐1009. 23333308 10.1016/j.molcel.2012.12.010 PMC3595374

[54] Clauson C , Schärer OD , Niedernhofer L . Advances in understanding the complex mechanisms of DNA interstrand cross‐link repair. Cold Spring Harb Perspect Biol. 2013;5(10):a012732. 24086043 10.1101/cshperspect.a012732 PMC4123742

[55] Smogorzewska A . Fanconi anemia: a paradigm for understanding DNA repair during replication. Blood. 2019;134(Supplement_1):SCI‐32.

[56] Karikkineth AC , Scheibye‐Knudsen M , Fivenson E , Croteau DL , Bohr VA . Cockayne syndrome: clinical features, model systems and pathways. Ageing Res Rev. 2017;33:3‐17. 27507608 10.1016/j.arr.2016.08.002 PMC5195851

[57] Hashimoto S , Egly JM . Trichothiodystrophy view from the molecular basis of DNA repair/transcription factor TFIIH. Hum Mol Genet. 2009;18(R2):R224‐R230. 19808800 10.1093/hmg/ddp390

[58] Fayyad N , Kobaisi F , Beal D , et al. Xeroderma pigmentosum C (XPC) Mutations in primary fibroblasts impair base excision repair pathway and increase oxidative DNA damage. Front Genet. 2020;11:561687. 33329698 10.3389/fgene.2020.561687 PMC7728722

[59] Suzumura H , Arisaka O . Cerebro‐oculo‐facio‐skeletal syndrome. Adv Exp Med Biol. 2010;685:210‐214. 20687508 10.1007/978-1-4419-6448-9_19

[60] Kaina B , Christmann M , Naumann S , Roos WP . MGMT: key node in the battle against genotoxicity, carcinogenicity and apoptosis induced by alkylating agents. DNA Repair. 2007;6(8):1079‐1099. 17485253 10.1016/j.dnarep.2007.03.008

[61] Hiddinga BI , Pauwels P , Janssens A , van Meerbeeck JP . O6‐methylguanine‐DNA methyltransferase (MGMT): a drugable target in lung cancer? Lung Cancer. 2017;107:91‐99. 27492578 10.1016/j.lungcan.2016.07.014

[62] Plummer R . Perspective on the pipeline of drugs being developed with modulation of DNA damage as a target. Clin Cancer Res. 2010;16(18):4527‐4531. 20823148 10.1158/1078-0432.CCR-10-0984

[63] Falck J , Coates J , Jackson SP . Conserved modes of recruitment of ATM, ATR and DNA‐PKcs to sites of DNA damage. Nature. 2005;434(7033):605‐611. 15758953 10.1038/nature03442

[64] Stewart GS , Panier S , Townsend K , et al. The RIDDLE syndrome protein mediates a ubiquitin‐dependent signaling cascade at sites of DNA damage. Cell. 2009;136(3):420‐434. 19203578 10.1016/j.cell.2008.12.042

[65] Huyen Y , Zgheib O , Ditullio RA , et al. Methylated lysine 79 of histone H3 targets 53BP1 to DNA double‐strand breaks. Nature. 2004;432(7015):406‐411. 15525939 10.1038/nature03114

[66] Stewart GS , Wang B , Bignell CR , Taylor AMR , Elledge SJ . MDC1 is a mediator of the mammalian DNA damage checkpoint. Nature. 2003;421(6926):961‐966. 12607005 10.1038/nature01446

[67] Gatei M , Sloper K , Sorensen C , et al. Ataxia‐telangiectasia‐mutated (ATM) and NBS1‐dependent phosphorylation of Chk1 on Ser‐317 in response to ionizing radiation. J Biol Chem. 2003;278(17):14806‐14811. 12588868 10.1074/jbc.M210862200

[68] Jazayeri A , Falck J , Lukas C , et al. ATM‐ and cell cycle‐dependent regulation of ATR in response to DNA double‐strand breaks. Nat Cell Biol. 2006;8(1):37‐45. 16327781 10.1038/ncb1337

[69] Stiff T , Walker SA , Cerosaletti K , et al. ATR‐dependent phosphorylation and activation of ATM in response to UV treatment or replication fork stalling. EMBO J. 2006;25(24):5775‐5782. 17124492 10.1038/sj.emboj.7601446 PMC1698893

[70] Zou L , Elledge SJ . Sensing DNA damage through ATRIP recognition of RPA‐ssDNA complexes. Science. 2003;300(5625):1542‐1548. 12791985 10.1126/science.1083430

[71] Cimprich KA , Cortez D . ATR: an essential regulator of genome integrity. Nat Rev Mol Cell Biol. 2008 Aug;9(8):616‐627. 18594563 10.1038/nrm2450 PMC2663384

[72] Zhou BBS , Bartek J . Targeting the checkpoint kinases: chemosensitization versus chemoprotection. Nat Rev Cancer. 2004;4(3):216‐225. 14993903 10.1038/nrc1296

[73] Pon JR , Marra MA . Driver and passenger mutations in cancer. Annu Rev Pathol. 2015;10:25‐50. 25340638 10.1146/annurev-pathol-012414-040312

[74] Rogozin IB , Goncearenco A , Lada AG , et al. DNA polymerase η mutational signatures are found in a variety of different types of cancer. Cell Cycle. 2018;17(3):348‐355. 29139326 10.1080/15384101.2017.1404208 PMC5914734

[75] Abdel‐Rahman MH , Sample KM , Pilarski R , et al. Whole exome sequencing identifies candidate genes associated with hereditary predisposition to uveal melanoma. Ophthalmology. 2020;127(5):668‐678. 32081490 10.1016/j.ophtha.2019.11.009 PMC7183432

[76] Pearl LH , Schierz AC , Ward SE , Al‐Lazikani B , Pearl FMG . Therapeutic opportunities within the DNA damage response. Nat Rev Cancer. 2015;15(3):166‐180. 25709118 10.1038/nrc3891

[77] O'Connor MJ . Targeting the DNA damage response in cancer. Mol Cell. 2015;60(4):547‐560. 26590714 10.1016/j.molcel.2015.10.040

[78] Khanna A . DNA damage in cancer therapeutics: a boon or a curse? Cancer Res. 2015;75(11):2133‐2138. 25931285 10.1158/0008-5472.CAN-14-3247

[79] Curtin NJ . DNA repair dysregulation from cancer driver to therapeutic target. Nat Rev Cancer. 2012;12(12):801‐817. 23175119 10.1038/nrc3399

[80] Huang R , Zhou PK . DNA damage repair: historical perspectives, mechanistic pathways and clinical translation for targeted cancer therapy. Sig Transduct Target Ther. 2021;6(1):1‐35. 10.1038/s41392-021-00648-7 PMC8266832 34238917

[81] Narayan V , Thompson EW , Demissei B , Ho JE , Januzzi JL , Ky B . Mechanistic biomarkers informative of both cancer and cardiovascular disease: JACC state‐of‐the‐art review. J Am Coll Cardiol. 2020;75(21):2726‐2737. 32466889 10.1016/j.jacc.2020.03.067 PMC7261288

[82] Siggens L , Figg N , Bennett M , Foo R . Nutrient deprivation regulates DNA damage repair in cardiomyocytes via loss of the base‐excision repair enzyme OGG1. FASEB J. 2012;26(5):2117‐2124. 22302830 10.1096/fj.11-197525 PMC3630495

[83] Sano M , Minamino T , Toko H , et al. p53‐induced inhibition of Hif‐1 causes cardiac dysfunction during pressure overload. Nature. 2007;446(7134):444‐448. 17334357 10.1038/nature05602

[84] Shukla PC , Singh KK , Quan A , et al. BRCA1 is an essential regulator of heart function and survival following myocardial infarction. Nat Commun. 2011;20(2):593. 10.1038/ncomms1601 PMC3247816 22186889

[85] Matsuoka S , Ballif BA , Smogorzewska A , et al. ATM and ATR substrate analysis reveals extensive protein networks responsive to DNA damage. Science. 2007;316(5828):1160‐1166. 17525332 10.1126/science.1140321

[86] Botto N , Rizza A , Colombo MG , et al. Evidence for DNA damage in patients with coronary artery disease. Mutat Res Genet Toxicol Environ Mutagen. 2001;493(1):23‐30. 10.1016/s1383-5718(01)00162-0 11516712

[87] Pilié PG , Tang C , Mills GB , Yap TA . State‐of‐the‐art strategies for targeting the DNA damage response in cancer. Nat Rev Clin Oncol. 2019;16(2):81‐104. 30356138 10.1038/s41571-018-0114-z PMC8327299

[88] Ait‐Aissa K , Blaszak SC , Beutner G , et al. Mitochondrial oxidative phosphorylation defect in the heart of subjects with coronary artery disease. Sci Rep. 2019;9(1):7623. 31110224 10.1038/s41598-019-43761-y PMC6527853

[89] Uryga A , Gray K , Bennett M . DNA damage and repair in vascular disease. Annu Rev Physiol. 2016;78(1):45‐66. 26442438 10.1146/annurev-physiol-021115-105127

[90] Sato M , Kadomatsu T , Miyata K , et al. The lncRNA Caren antagonizes heart failure by inactivating DNA damage response and activating mitochondrial biogenesis. Nat Commun. 2021;12(1):2529. 33953175 10.1038/s41467-021-22735-7 PMC8099897

[91] Gray K , Kumar S , Figg N , et al. Effects of DNA damage in smooth muscle cells in atherosclerosis. Circ Res. 2015;116(5):816‐826. 25524056 10.1161/CIRCRESAHA.116.304921

[92] Haemmig S , Yang D , Sun X , et al. Long noncoding RNA SNHG12 integrates a DNA‐PK‐mediated DNA damage response and vascular senescence. Sci Transl Med. 2020;12(531):eaaw1868. 32075942 10.1126/scitranslmed.aaw1868

[93] Caldecott KW . Single‐strand break repair and genetic disease. Nat Rev Genet. 2008;9(8):619‐631. 18626472 10.1038/nrg2380

[94] Higo T , Naito AT , Sumida T , et al. DNA single‐strand break‐induced DNA damage response causes heart failure. Nat Commun. 2017;24(8):15104. 10.1038/ncomms15104 PMC5413978 28436431

[95] Durik M , Kavousi M , van der Pluijm I , et al. Nucleotide excision DNA repair is associated with age‐related vascular dysfunction. Circulation. 2012;126(4):468‐478. 22705887 10.1161/CIRCULATIONAHA.112.104380 PMC3430727

[96] Ishida T , Ishida M , Tashiro S , Yoshizumi M , Kihara Y . Role of DNA damage in cardiovascular disease. Circ J. 2014;78(1):42‐50. 24334614 10.1253/circj.cj-13-1194

[97] Su Y , Swift M . Mortality rates among carriers of ataxia‐telangiectasia mutant alleles. Ann Intern Med. 2000;133(10):770‐778. 11085839 10.7326/0003-4819-133-10-200011210-00009

[98] Swift M , Chase C . Cancer and cardiac deaths in obligatory ataxia‐telangiectasia heterozygotes. Lancet. 1983;1(8332):1049‐1050. 10.1016/s0140-6736(83)92678-8 6133091

[99] Jeon BH , Gupta G , Park YC , et al. Apurinic/apyrimidinic endonuclease 1 regulates endothelial NO production and vascular tone. Circ Res. 2004;95(9):902‐910. 15472121 10.1161/01.RES.0000146947.84294.4c

[100] Nakayama T , Sato W , Yoshimura A , et al. Endothelial von willebrand factor release due to eNOS deficiency predisposes to thrombotic microangiopathy in mouse aging kidney. Am J Pathol. 2010;176(5):2198‐2208. 20363914 10.2353/ajpath.2010.090316 PMC2861085

[101] Erusalimsky JD . Vascular endothelial senescence: from mechanisms to pathophysiology. J Appl Physiol. 2009;106(1):326‐332. 19036896 10.1152/japplphysiol.91353.2008 PMC2636933

[102] Fackenthal JD , Olopade OI . Breast cancer risk associated with BRCA1 and BRCA2 in diverse populations. Nat Rev Cancer. 2007;7(12):937‐948. 18034184 10.1038/nrc2054

[103] Mai PL , Chatterjee N , Hartge P , et al. Potential excess mortality in BRCA1/2 mutation carriers beyond breast, ovarian, prostate, and pancreatic cancers, and melanoma. PLoS One. 2009;4(3):e4812. 19277124 10.1371/journal.pone.0004812 PMC2652075

[104] Bordeleau L , Lipscombe L , Lubinski J , et al. Diabetes and breast cancer among women with BRCA1 and BRCA2 mutations. Cancer. 2011;117(9):1812‐1818. 21509758 10.1002/cncr.25595 PMC3413077

[105] Mocanu MM , Yellon DM . p53 down‐regulation: a new molecular mechanism involved in ischaemic preconditioning. FEBS Lett. 2003;555(2):302‐306. 14644432 10.1016/s0014-5793(03)01260-2

[106] Vousden KH , Lane DP . p53 in health and disease. Nat Rev Mol Cell Biol. 2007;8(4):275‐283. 17380161 10.1038/nrm2147

[107] Hakem R , de la Pompa JL , Mak TW . Developmental studies of Brca1 and Brca2 knock‐out mice. J Mammary Gland Biol Neoplasia. 1998;3(4):431‐445. 10819537 10.1023/a:1018792200700

[108] Matsusaka H , Ide T , Matsushima S , et al. Targeted deletion of p53 prevents cardiac rupture after myocardial infarction in mice. Cardiovasc Res. 2006;70(3):457‐465. 16533502 10.1016/j.cardiores.2006.02.001

[109] Zhang Y , Köhler K , Xu J , et al. Inhibition of p53 after acute myocardial infarction: reduction of apoptosis is counteracted by disturbed scar formation and cardiac rupture. J Mol Cell Cardiol. 2011;50(3):471‐478. 21074539 10.1016/j.yjmcc.2010.11.006

[110] Naito AT , Okada S , Minamino T , et al. Promotion of CHIP‐mediated p53 degradation protects the heart from ischemic injury. Circ Res. 2010;106(11):1692‐1702. 20413784 10.1161/CIRCRESAHA.109.214346

[111] Bialik S , Geenen DL , Sasson IE , et al. Myocyte apoptosis during acute myocardial infarction in the mouse localizes to hypoxic regions but occurs independently of p53. J Clin Invest. 1997;100(6):1363‐1372. 9294101 10.1172/JCI119656 PMC508314

[112] Lane DP . Cancer. p53, guardian of the genome. Nature. 1992;358(6381):15‐16. 1614522 10.1038/358015a0

[113] Levine AJ . p53: 800 million years of evolution and 40 years of discovery. Nat Rev Cancer. 2020;20(8):471‐480. 32404993 10.1038/s41568-020-0262-1

[114] Kandoth C , McLellan MD , Vandin F , et al. Mutational landscape and significance across 12 major cancer types. Nature. 2013;502(7471):333‐339. 24132290 10.1038/nature12634 PMC3927368

[115] Malkin D , Li FP , Strong LC , et al. Germ line p53 mutations in a familial syndrome of breast cancer, sarcomas, and other neoplasms. Science. 1990;250(4985):1233‐1238. 1978757 10.1126/science.1978757

[116] Zhu G , Pan C , Bei JX , et al. Mutant p53 in cancer progression and targeted therapies. Front Oncol. 2020;6(10):595187. 10.3389/fonc.2020.595187 PMC7677253 33240819

[117] Brosh R , Rotter V . When mutants gain new powers: news from the mutant p53 field. Nat Rev Cancer. 2009;9(10):701‐713. 19693097 10.1038/nrc2693

[118] D'Orazi G , Cirone M . Mutant p53 and cellular stress pathways: a criminal alliance that promotes cancer progression. Cancers. 2019;11(5):614. 31052524 10.3390/cancers11050614 PMC6563084

[119] Zhang Q , He X , Chen L , et al. Synergistic regulation of p53 by Mdm2 and Mdm4 is critical in cardiac endocardial cushion morphogenesis during heart development. J Pathol. 2012;228(3):416‐428. 22821713 10.1002/path.4077

[120] Mak TW , Hauck L , Grothe D , Billia F . p53 regulates the cardiac transcriptome. Proc Natl Acad Sci USA. 2017;114(9):2331‐2336. 28193895 10.1073/pnas.1621436114 PMC5338492

[121] Chatterjee A , Mir SA , Dutta D , Mitra A , Pathak K , Sarkar S . Analysis of p53 and NF‐κB signaling in modulating the cardiomyocyte fate during hypertrophy. J Cell Physiol. 2011;226(10):2543‐2554. 21792911 10.1002/jcp.22599

[122] Nakamura H , Matoba S , Iwai‐Kanai E , et al. p53 promotes cardiac dysfunction in diabetic mellitus caused by excessive mitochondrial respiration‐mediated reactive oxygen species generation and lipid accumulation. Circ Heart Fail. 2012;5(1):106‐115. 22075967 10.1161/CIRCHEARTFAILURE.111.961565

[123] Mercer J , Figg N , Stoneman V , Braganza D , Bennett MR . Endogenous p53 protects vascular smooth muscle cells from apoptosis and reduces atherosclerosis in ApoE knockout mice. Circ Res. 2005;96(6):667‐674. 15746445 10.1161/01.RES.0000161069.15577.ca

[124] Xu T , Ding W , Ao X , et al. ARC regulates programmed necrosis and myocardial ischemia/reperfusion injury through the inhibition of mPTP opening. Redox Biol. 2019;1(20):414‐426. 10.1016/j.redox.2018.10.023 PMC6230922 30415165

[125] Beckerman R , Prives C . Transcriptional regulation by P53. Cold Spring Harb Perspect Biol. 2010;2(8):a000935. 20679336 10.1101/cshperspect.a000935 PMC2908772

[126] Vousden KH , Prives C . Blinded by the light: the growing complexity of p53. Cell. 2009;137(3):413‐431. 19410540 10.1016/j.cell.2009.04.037

[127] Manfredi JJ . The Mdm2–p53 relationship evolves: Mdm2 swings both ways as an oncogene and a tumor suppressor. Genes Dev. 2010;24(15):1580‐1589. 20679392 10.1101/gad.1941710 PMC2912554

[128] Song H , Conte JV , Foster AH , McLaughlin JS , Wei C . Increased p53 protein expression in human failing myocardium. J Heart Lung Transplant. 1999;18(8):744‐749. 10512520 10.1016/s1053-2498(98)00039-4

[129] Munshi N , Fernandis AZ , Cherla RP , Park IW , Ganju RK . Lipopolysaccharide‐induced apoptosis of endothelial cells and its inhibition by vascular endothelial growth factor. J Immunol. 2002;168(11):5860‐5866. 12023390 10.4049/jimmunol.168.11.5860

[130] Gogiraju R , Xu X , Bochenek ML , et al. Endothelial p53 deletion improves angiogenesis and prevents cardiac fibrosis and heart failure induced by pressure overload in mice. J Am Heart Assoc. 2015;4(2):e001770. 25713289 10.1161/JAHA.115.001770 PMC4345879

[131] Yokoyama M , Okada S , Nakagomi A , et al. Inhibition of endothelial p53 improves metabolic abnormalities related to dietary obesity. Cell Rep. 2014;7(5):1691‐1703. 24857662 10.1016/j.celrep.2014.04.046

[132] Kumar A , Kim CS , Hoffman TA , et al. p53 impairs endothelial function by transcriptionally repressing kruppel‐like factor 2. Arterioscler Thromb Vasc Biol. 2011;31(1):133‐141. 20947822 10.1161/ATVBAHA.110.215061 PMC3064482

[133] Lukin DJ , Carvajal LA , Liu WJ , Resnick‐Silverman L , Manfredi JJ . p53 Promotes cell survival due to the reversibility of its cell‐cycle checkpoints. Mol Cancer Res. 2015;13(1):16‐28. 25158956 10.1158/1541-7786.MCR-14-0177 PMC4312522

[134] Panta S , Yamakuchi M , Shimizu T , et al. Low grade inflammation inhibits VEGF induced HUVECs migration in p53 dependent manner. Biochem Biophys Res Commun. 2017;483(2):803‐809. 27998768 10.1016/j.bbrc.2016.12.096

[135] Guevara NV , Kim HS , Antonova EI , Chan L . The absence of p53 accelerates atherosclerosis by increasing cell proliferation in vivo. Nat Med. 1999;5(3):335‐339. 10086392 10.1038/6585

[136] van Vlijmen BJ , Gerritsen G , Franken AL , et al. Macrophage p53 deficiency leads to enhanced atherosclerosis in APOE*3‐Leiden transgenic mice. Circ Res. 2001;88(8):780‐786. 11325869 10.1161/hh0801.089261

[137] Heo KS , Lee H , Nigro P , et al. PKCζ mediates disturbed flow‐induced endothelial apoptosis via p53 SUMOylation. J Cell Biol. 2011;193(5):867‐884. 21624955 10.1083/jcb.201010051 PMC3105539

[138] Kim KS , Kang KW , Seu YB , Baek SH , Kim JR . Interferon‐γ induces cellular senescence through p53‐dependent DNA damage signaling in human endothelial cells. Mech Ageing Dev. 2009;130(3):179‐188. 19071156 10.1016/j.mad.2008.11.004

[139] Donato AJ , Magerko KA , Lawson BR , Durrant JR , Lesniewski LA , Seals DR . SIRT‐1 and vascular endothelial dysfunction with ageing in mice and humans. J Physiol. 2011;589(18):4545‐4554. 21746786 10.1113/jphysiol.2011.211219 PMC3208223

[140] Luo J , Nikolaev AY , Imai S ichiro, Chen D , Su F , Shiloh A , et al. Negative control of p53 by Sir2α promotes cell survival under stress. Cell 2001 107(2):137–48. 11672522 10.1016/s0092-8674(01)00524-4

[141] Vaziri H , Dessain SK , Eaton EN , et al. hSIR2SIRT1 functions as an NAD‐dependent p53 deacetylase. Cell. 2001;107(2):149‐159. 11672523 10.1016/s0092-8674(01)00527-x

[142] Srinivas US , Tan BWQ , Vellayappan BA , Jeyasekharan AD . ROS and the DNA damage response in cancer. Redox Biol. 2019;1(25):101084. 10.1016/j.redox.2018.101084 PMC6859528 30612957

[143] Conklin KA . Chemotherapy‐associated oxidative stress: impact on chemotherapeutic effectiveness. Integr Cancer Ther. 2004;3(4):294‐300. 15523100 10.1177/1534735404270335

[144] Marullo R , Werner E , Degtyareva N , et al. Cisplatin induces a mitochondrial‐ROS response that contributes to cytotoxicity depending on mitochondrial redox status and bioenergetic functions. PLoS One. 2013;8(11):e81162. 24260552 10.1371/journal.pone.0081162 PMC3834214

[145] Kunsch C , Medford RM . Oxidative stress as a regulator of gene expression in the vasculature. Circ Res. 1999;85(8):753‐766. 10521248 10.1161/01.res.85.8.753

[146] Irani K . Oxidant signaling in vascular cell growth, death, and survival: a review of the roles of reactive oxygen species in smooth muscle and endothelial cell mitogenic and apoptotic signaling. Circ Res. 2000;87(3):179‐183. 10926866 10.1161/01.res.87.3.179

[147] Salehi F , Behboudi H , Kavoosi G , Ardestani SK . Oxidative DNA damage induced by ROS‐modulating agents with the ability to target DNA: a comparison of the biological characteristics of citrus pectin and apple pectin. Sci Rep. 2018;8(1):13902. 30224635 10.1038/s41598-018-32308-2 PMC6141541

[148] Basta G , Schmidt AM , De Caterina R . Advanced glycation end products and vascular inflammation: implications for accelerated atherosclerosis in diabetes. Cardiovasc Res. 2004;63(4):582‐592. 15306213 10.1016/j.cardiores.2004.05.001

[149] Simon AS , Chithra V , Vijayan A , Dinesh RD , Vijayakumar T . Altered DNA repair, oxidative stress and antioxidant status in coronary artery disease. J Biosci. 2013;38(2):385‐389. 23660673 10.1007/s12038-013-9313-z

[150] Ballinger SW , Patterson C , Knight‐Lozano CA , et al. Mitochondrial integrity and function in atherogenesis. Circulation. 2002 Jul 30;106(5):544‐549. 12147534 10.1161/01.cir.0000023921.93743.89

[151] Cannan WJ , Tsang BP , Wallace SS , Pederson DS . Nucleosomes suppress the formation of double‐strand DNA breaks during attempted base excision repair of clustered oxidative damages*. Journal of Biological Chemistry. 2014;289(29):19881‐19893. 24891506 10.1074/jbc.M114.571588 PMC4106309

[152] Botto N , Masetti S , Petrozzi L , et al. Elevated levels of oxidative DNA damage in patients with coronary artery disease. Coron Artery Dis. 2002;13(5):269‐274. 12394651 10.1097/00019501-200208000-00004

[153] Martinet W , Knaapen MW , De Meyer GR , Herman AG , Kockx MM . Elevated levels of oxidative DNA damage and DNA repair enzymes in human atherosclerotic plaques. Circulation. 2002;106(8):927‐932. 12186795 10.1161/01.cir.0000026393.47805.21

[154] Singh KK , Shukla PC , Quan A , et al. BRCA1 is a novel target to improve endothelial dysfunction and retard atherosclerosis. J Thorac Cardiovasc Surg. 2013;146(4):949‐960.e4. 23415688 10.1016/j.jtcvs.2012.12.064

[155] Singh S , Nguyen H , Michels D , et al. BReast CAncer susceptibility gene 2 deficiency exacerbates oxidized LDL‐induced DNA damage and endothelial apoptosis. Physiol Rep. 2020;8(13):e14481. 32638521 10.14814/phy2.14481 PMC7340845

[156] Matthews C , Gorenne I , Scott S , et al. Vascular smooth muscle cells undergo telomere‐based senescence in human atherosclerosis. Circ Res. 2006;99(2):156‐164. 16794190 10.1161/01.RES.0000233315.38086.bc

[157] Durand E , Scoazec A , Lafont A , et al. In vivo induction of endothelial apoptosis leads to vessel thrombosis and endothelial denudation: a clue to the understanding of the mechanisms of thrombotic plaque erosion. Circulation. 2004;109(21):2503‐2506. 15148270 10.1161/01.CIR.0000130172.62481.90

[158] von der Thüsen JH , van Vlijmen BJM , Hoeben RC , et al. Induction of atherosclerotic plaque rupture in apolipoprotein E‐/‐ mice after adenovirus‐mediated transfer of p53. Circulation. 2002 Apr 30;105(17):2064‐2070. 11980686 10.1161/01.cir.0000015502.97828.93

[159] Warboys CM , de Luca A , Amini N , et al. Disturbed flow promotes endothelial senescence via a p53‐dependent pathway. Arterioscler Thromb Vasc Biol. 2014;34(5):985‐995. 24651677 10.1161/ATVBAHA.114.303415

[160] Khanna AK . Enhanced susceptibility of cyclin kinase inhibitor p21 knockout mice to high fat diet induced atherosclerosis. J Biomed Sci. 2009;16(1):66. 19604372 10.1186/1423-0127-16-66 PMC2720941

[161] Mercer JR , Cheng KK , Figg N , et al. DNA damage links mitochondrial dysfunction to atherosclerosis and the metabolic syndrome. Circ Res. 2010;107(8):1021‐1031. 20705925 10.1161/CIRCRESAHA.110.218966 PMC2982998

[162] Martinet W , Knaapen MW , De Meyer GR , Herman AG , Kockx MM . Oxidative DNA damage and repair in experimental atherosclerosis are reversed by dietary lipid lowering. Circ Res. 2001;88(7):733‐739. 11304497 10.1161/hh0701.088684

[163] Lüscher TF , Barton M . Biology of the endothelium. Clin Cardiol. 1997;20(11 Suppl 2):II‐3‐10. 10.1002/j.1932-8737.1997.tb00006.x PMC7509692 9422846

[164] Yang YM , Huang A , Kaley G , Sun D . eNOS uncoupling and endothelial dysfunction in aged vessels. Am J Physiol Heart Circ Physiol. 2009;297(5):H1829‐H1836. 19767531 10.1152/ajpheart.00230.2009 PMC2781386

[165] Moncada S , Palmer RM , Higgs EA . Nitric oxide: physiology, pathophysiology, and pharmacology. Pharmacol Rev. 1991;43(2):109‐142. 1852778

[166] Rudic RD , Shesely EG , Maeda N , Smithies O , Segal SS , Sessa WC . Direct evidence for the importance of endothelium‐derived nitric oxide in vascular remodeling. J Clin Invest. 1998;101(4):731‐736. 9466966 10.1172/JCI1699 PMC508619

[167] Huang PL , Huang Z , Mashimo H , et al. Hypertension in mice lacking the gene for endothelial nitric oxide synthase. Nature. 1995;377(6546):239‐242. 7545787 10.1038/377239a0

[168] Murohara T , Asahara T , Silver M , et al. Nitric oxide synthase modulates angiogenesis in response to tissue ischemia. J Clin Invest. 1998;101(11):2567‐2578. 9616228 10.1172/JCI1560 PMC508846

[169] Freedman JE , Sauter R , Battinelli EM , et al. Deficient platelet‐derived nitric oxide and enhanced hemostasis in mice lacking the NOSIII gene. Circ Res. 1999;84(12):1416‐1421. 10381894 10.1161/01.res.84.12.1416

[170] Tai SC , Robb GB , Marsden PA . Endothelial nitric oxide synthase: a new paradigm for gene regulation in the injured blood vessel. Arterioscler Thromb Vasc Biol. 2004;24(3):405‐412. 14656742 10.1161/01.ATV.0000109171.50229.33

[171] Kushwaha S , Vikram A , Trivedi PP , Jena GB . Alkaline, Endo III and FPG modified comet assay as biomarkers for the detection of oxidative DNA damage in rats with experimentally induced diabetes. Mutat Res. 2011;726(2):242‐250. 22015262 10.1016/j.mrgentox.2011.10.004

[172] Quagliaro L , Piconi L , Assaloni R , Martinelli L , Motz E , Ceriello A . Intermittent high glucose enhances apoptosis related to oxidative stress in human umbilical vein endothelial cells: the role of protein kinase C and NAD(P)H‐oxidase activation. Diabetes. 2003;52(11):2795‐2804. 14578299 10.2337/diabetes.52.11.2795

[173] Takao T , Horino T , Kagawa T , et al. Possible involvement of intracellular angiotensin II receptor in high‐glucose‐induced damage in renal proximal tubular cells. J Nephrol. 2011;24(2):218‐224. 20890878 10.5301/jn.2010.5785

[174] Stopper H , Schinzel R , Sebekova K , Heidland A . Genotoxicity of advanced glycation end products in mammalian cells. Cancer Lett. 2003;190(2):151‐156. 12565169 10.1016/s0304-3835(02)00626-2

[175] Schupp N , Schinzel R , Heidland A , Stopper H . Genotoxicity of advanced glycation end products: involvement of oxidative stress and of angiotensin II type 1 receptors. Ann N Y Acad Sci. 2005;1043:685‐695. 16037294 10.1196/annals.1333.079

[176] Fukami K , Yamagishi S ichi, Kaifu K , Matsui T , Kaida Y , Ueda S , et al. Telmisartan inhibits AGE‐induced podocyte damage and detachment. Microvasc Res 2013 88:79–83. 23648312 10.1016/j.mvr.2013.04.006

[177] Mizutani K , Ikeda K , Nishikata T , Yamori Y . Phytoestrogens attenuate oxidative DNA damage in vascular smooth muscle cells from stroke‐prone spontaneously hypertensive rats. J Hypertens. 2000;18(12):1833‐1840. 11132608 10.1097/00004872-200018120-00018

[178] Orie NN , Zidek W , Tepel M . Reactive oxygen species in essential hypertension and non‐insulin‐dependent diabetes mellitus. Am J Hypertens. 1999;12(12 Pt 1‐2):1169‐1174. 10619578 10.1016/s0895-7061(99)00129-6

[179] Seghrouchni I , Drai J , Bannier E , et al. Oxidative stress parameters in type I, type II and insulin‐treated type 2 diabetes mellitus; insulin treatment efficiency. Clin Chim Acta. 2002;321(1–2):89‐96. 12031597 10.1016/s0009-8981(02)00099-2

[180] Simone S , Gorin Y , Velagapudi C , Abboud HE , Habib SL . Mechanism of oxidative DNA damage in diabetes: tuberin inactivation and downregulation of DNA repair enzyme 8‐oxo‐7,8‐dihydro‐2'‐deoxyguanosine‐DNA glycosylase. Diabetes. 2008;57(10):2626‐2636. 18599524 10.2337/db07-1579 PMC2551671

[181] Scully R , Livingston DM . In search of the tumour‐suppressor functions of BRCA1 and BRCA2. Nature. 2000;408(6811):429‐432. 11100717 10.1038/35044000 PMC2981135

[182] Blasiak J , Arabski M , Krupa R , et al. DNA damage and repair in type 2 diabetes mellitus. Mutat Res. 2004;554(1–2):297‐304. 15450427 10.1016/j.mrfmmm.2004.05.011

[183] Farhangkhoee H , Khan ZA , Barbin Y , Chakrabarti S . Glucose‐induced up‐regulation of CD36 mediates oxidative stress and microvascular endothelial cell dysfunction. Diabetologia. 2005;48(7):1401‐1410. 15915335 10.1007/s00125-005-1801-8

[184] Quagliaro L , Piconi L , Assaloni R , et al. Intermittent high glucose enhances ICAM‐1, VCAM‐1 and E‐selectin expression in human umbilical vein endothelial cells in culture: the distinct role of protein kinase C and mitochondrial superoxide production. Atherosclerosis. 2005;183(2):259‐267. 16285992 10.1016/j.atherosclerosis.2005.03.015

[185] Pathak N , Khandelwal S . Impact of cadmium in T lymphocyte subsets and cytokine expression: differential regulation by oxidative stress and apoptosis. Biometals. 2008;21(2):179‐187. 17641822 10.1007/s10534-007-9106-7

[186] Oumouna‐Benachour K , Hans CP , Suzuki Y , et al. Poly(ADP‐ribose) polymerase inhibition reduces atherosclerotic plaque size and promotes factors of plaque stability in apolipoprotein E‐deficient mice: effects on macrophage recruitment, nuclear factor‐kappaB nuclear translocation, and foam cell death. Circulation. 2007;115(18):2442‐2450. 17438151 10.1161/CIRCULATIONAHA.106.668756

[187] Godschalk RWL , Albrecht C , Curfs DMJ , et al. Decreased levels of lipid peroxidation‐induced DNA damage in the onset of atherogenesis in apolipoprotein E deficient mice. Mutat Res. 2007;621(1–2):87‐94. 17418875 10.1016/j.mrfmmm.2007.02.012

[188] Andreassi MG , Botto N . DNA damage as a new emerging risk factor in atherosclerosis. Trends Cardiovasc Med. 2003;13(7):270‐275. 14522466 10.1016/s1050-1738(03)00109-9

[189] Harangi M , Remenyik EE , Seres I , Varga Z , Katona E , Paragh G . Determination of DNA damage induced by oxidative stress in hyperlipidemic patients. Mutat Res. 2002;513(1–2):17‐25. 11719086 10.1016/s1383-5718(01)00285-6

[190] Demirbag R , Yilmaz R , Kocyigit A . Relationship between DNA damage, total antioxidant capacity and coronary artery disease. Mutat Res. 2005;570(2):197‐203. 15708578 10.1016/j.mrfmmm.2004.11.003

[191] Borghini A , Cervelli T , Galli A , Andreassi MG . DNA modifications in atherosclerosis: from the past to the future. Atherosclerosis. 2013;230(2):202‐209. 24075745 10.1016/j.atherosclerosis.2013.07.038

[192] Van Gaal LF , Mertens IL , De Block CE . Mechanisms linking obesity with cardiovascular disease. Nature. 2006;444(7121):875‐880. 17167476 10.1038/nature05487

[193] Shimizu I , Yoshida Y , Suda M , Minamino T . DNA damage response and metabolic disease. Cell Metab. 2014;20(6):967‐977. 25456739 10.1016/j.cmet.2014.10.008

[194] Włodarczyk M , Jabłonowska‐Lietz B , Olejarz W , Nowicka G . Anthropometric and dietary factors as predictors of DNA damage in obese women. Nutrients. 2018;10(5):E578. 10.3390/nu10050578 PMC5986458 29738492

[195] Sancar A . Excision repair in mammalian cells. J Biol Chem. 1995;270(27):15915‐15918. 7608140 10.1074/jbc.270.27.15915

[196] Bukhari SA , Rajoka MI , Ibrahim Z , Jalal F , Rana SM , Nagra SA . Oxidative stress elevated DNA damage and homocysteine level in normal pregnant women in a segment of Pakistani population. Mol Biol Rep. 2011;38(4):2703‐2710. 21107731 10.1007/s11033-010-0413-7

[197] Setayesh T , Nersesyan A , Mišík M , et al. Impact of obesity and overweight on DNA stability: few facts and many hypotheses. Mutat Res Rev Mutat Res. 2018;777:64‐91. 30115431 10.1016/j.mrrev.2018.07.001

[198] Han CY , Umemoto T , Omer M , et al. NADPH oxidase‐derived reactive oxygen species increases expression of monocyte chemotactic factor genes in cultured adipocytes. J Biol Chem. 2012;287(13):10379‐10393. 22287546 10.1074/jbc.M111.304998 PMC3322984

[199] Weisberg SP , McCann D , Desai M , Rosenbaum M , Leibel RL , Ferrante AW . Obesity is associated with macrophage accumulation in adipose tissue. J Clin Invest. 2003;112(12):1796‐1808. 14679176 10.1172/JCI19246 PMC296995

[200] Gao CL , Zhu C , Zhao YP , et al. Mitochondrial dysfunction is induced by high levels of glucose and free fatty acids in 3T3‐L1 adipocytes. Mol Cell Endocrinol. 2010;320(1–2):25‐33. 20144685 10.1016/j.mce.2010.01.039

[201] Heo JW , No MH , Park DH , et al. Effects of exercise on obesity‐induced mitochondrial dysfunction in skeletal muscle. Korean J Physiol Pharmacol. 2017;21(6):567‐577. 29200899 10.4196/kjpp.2017.21.6.567 PMC5709473

[202] Fehsel K , Kolb‐Bachofen V , Kolb H . Analysis of TNF alpha‐induced DNA strand breaks at the single cell level. Am J Pathol. 1991;139(2):251‐254. 1867316 PMC1886076

[203] Arango Duque G , Descoteaux A . Macrophage cytokines: involvement in immunity and infectious diseases. Front Immunol. 2014;5:491. 25339958 10.3389/fimmu.2014.00491 PMC4188125

[204] Rastogi S , Boylan M , Wright EG , Coates PJ . Interactions of apoptotic cells with macrophages in radiation‐induced bystander signaling. Radiat Res. 2013;179(2):135‐145. 23237586 10.1667/RR2969.1

[205] Speed N , Blair IA . Cyclooxygenase‐ and lipoxygenase‐mediated DNA damage. Cancer Metastasis Rev. 2011;30(3–4):437‐447. 22009064 10.1007/s10555-011-9298-8 PMC3237763

[206] Tyson J , Caple F , Spiers A , et al. Inter‐individual variation in nucleotide excision repair in young adults: effects of age, adiposity, micronutrient supplementation and genotype. Br J Nutr. 2009;101(9):1316‐1323. 18838045 10.1017/S0007114508076265

[207] Scarpato R , Verola C , Fabiani B , Bianchi V , Saggese G , Federico G . Nuclear damage in peripheral lymphocytes of obese and overweight Italian children as evaluated by the gamma‐H2AX focus assay and micronucleus test. FASEB J. 2011;25(2):685‐693. 21068397 10.1096/fj.10-168427

[208] Liu RH , Hotchkiss JH . Potential genotoxicity of chronically elevated nitric oxide: a review. Mutat Res. 1995;339(2):73‐89. 7791803 10.1016/0165-1110(95)90004-7

[209] McAdam E , Brem R , Karran P . Oxidative stress‐induced protein damage inhibits DNA repair and determines mutation risk and therapeutic efficacy. Mol Cancer Res. 2016;14(7):612‐622. 27106867 10.1158/1541-7786.MCR-16-0053 PMC4955916

[210] Barouch LA , Gao D , Chen L , et al. Cardiac myocyte apoptosis is associated with increased DNA damage and decreased survival in murine models of obesity. Circulation Research. 2006;98(1):119‐124. 16339484 10.1161/01.RES.0000199348.10580.1d

[211] Kalogeris T , Baines CP , Krenz M , Korthuis RJ . Cell biology of ischemia/reperfusion injury. Int Rev Cell Mol Biol. 2012;298:229‐317. 22878108 10.1016/B978-0-12-394309-5.00006-7 PMC3904795

[212] Tran DH , Wang ZV . Glucose metabolism in cardiac hypertrophy and heart failure. J Am Heart Assoc. 2019;8(12):e012673. 31185774 10.1161/JAHA.119.012673 PMC6645632

[213] Yellon DM , Hausenloy DJ . Myocardial reperfusion injury. New England Journal of Medicine. 2007 Sep 13;357(11):1121‐1135. 17855673 10.1056/NEJMra071667

[214] Badalzadeh R , Azimi A , Alihemmati A , Yousefi B . Chronic type‐I diabetes could not impede the anti‐inflammatory and anti‐apoptotic effects of combined postconditioning with ischemia and cyclosporine A in myocardial reperfusion injury. J Physiol Biochem. 2017;73(1):111‐120. 27771871 10.1007/s13105-016-0530-4

[215] Feyzizadeh S , Javadi A , Badalzadeh R , Vafaee MS . Signaling mediators modulated by cardioprotective interventions in healthy and diabetic myocardium with ischaemia–reperfusion injury. Eur J Prev Cardiol. 2018;25(14):1463‐1481. 29442529 10.1177/2047487318756420

[216] Gao Q , Deng H , Li H , et al. Glycolysis and fatty acid β‐oxidation, which one is the culprit of ischemic reperfusion injury? Int J Clin Exp Med. 2018;11(1):59‐68.

[217] Baxter P , Chen Y , Xu Y , Swanson RA . Mitochondrial dysfunction induced by nuclear poly(ADP‐Ribose) polymerase‐1: a treatable cause of cell death in stroke. Transl Stroke Res. 2014;5(1):136‐144. 24323707 10.1007/s12975-013-0283-0 PMC4034530

[218] Di Lisa F , Menabò R , Canton M , Barile M , Bernardi P . Opening of the mitochondrial permeability transition pore causes depletion of mitochondrial and cytosolic NAD+ and is a causative event in the death of myocytes in postischemic reperfusion of the heart. J Biol Chem. 2001;276(4):2571‐2575. 11073947 10.1074/jbc.M006825200

[219] Singh CK , Chhabra G , Ndiaye MA , Garcia‐Peterson LM , Mack NJ , Ahmad N . The role of sirtuins in antioxidant and redox signaling. Antioxid Redox Signal. 2018;28(8):643‐661. 28891317 10.1089/ars.2017.7290 PMC5824489

[220] Matsushima S , Sadoshima J . The role of sirtuins in cardiac disease. Am J Physiol Heart Circ Physiol. 2015;309(9):H1375‐H1389. 26232232 10.1152/ajpheart.00053.2015 PMC4666968

[221] Becatti M , Taddei N , Cecchi C , Nassi N , Nassi PA , Fiorillo C . SIRT1 modulates MAPK pathways in ischemic‐reperfused cardiomyocytes. Cell Mol Life Sci. 2012;69(13):2245‐2260. 22311064 10.1007/s00018-012-0925-5 PMC11114949

[222] Zhang J , Ren D , Fedorova J , He Z , Li J . SIRT1/SIRT3 modulates redox homeostasis during ischemia/reperfusion in the aging heart. Antioxidants. 2020;9(9):858. 32933202 10.3390/antiox9090858 PMC7556005

[223] Hosseini L , Vafaee MS , Mahmoudi J , Badalzadeh R . Nicotinamide adenine dinucleotide emerges as a therapeutic target in aging and ischemic conditions. Biogerontology. 2019;20(4):381‐395. 30838484 10.1007/s10522-019-09805-6

[224] Wu L , Xiong X , Wu X , et al. Targeting oxidative stress and inflammation to prevent ischemia‐reperfusion injury. Front Mol Neurosci. 2020;13:28. 32194375 10.3389/fnmol.2020.00028 PMC7066113

[225] Inafuku H , Kuniyoshi Y , Yamashiro S , et al. Determination of oxidative stress and cardiac dysfunction after ischemia/reperfusion injury in isolated rat hearts. Ann Thorac Cardiovasc Surg. 2013;19(3):186‐194. 22971810 10.5761/atcs.oa.12.01896

[226] Cordis GA , Maulik G , Bagchi D , Riedel W , Das DK . Detection of oxidative DNA damage to ischemic reperfused rat hearts by 8‐hydroxydeoxyguanosine formation. J Mol Cell Cardiol. 1998;30(10):1939‐1944. 9799648 10.1006/jmcc.1998.0752

[227] Shukla PC , Singh KK , Yanagawa B , Teoh H , Verma S . DNA damage repair and cardiovascular diseases. Can J Cardiol. 2010;1(26):13A‐16A. 10.1016/s0828-282x(10)71055-2 20386754

[228] Ko T , Fujita K , Nomura S , et al. Quantification of DNA damage in heart tissue as a novel prediction tool for therapeutic prognosis of patients with dilated cardiomyopathy. JACC Basic Transl Sci. 2019;4(6):670‐680. 31709317 10.1016/j.jacbts.2019.05.010 PMC6834953

[229] Singh KK , Shukla PC , Quan A , et al. BRCA2 protein deficiency exaggerates doxorubicin‐induced cardiomyocyte apoptosis and cardiac failure. J Biol Chem. 2012;287(9):6604‐6614. 22157755 10.1074/jbc.M111.292664 PMC3325595

[230] Yndestad A , Neurauter CG , Øie E , et al. Up‐regulation of myocardial DNA base excision repair activities in experimental heart failure. Mutat Res Fundam Mol Mech Mutagen. 2009;666(1):32‐38. 10.1016/j.mrfmmm.2009.03.008 19481677

[231] Xiao CY , Chen M , Zsengellér Z , et al. Poly(ADP‐ribose) polymerase promotes cardiac remodeling, contractile failure, and translocation of apoptosis‐inducing factor in a murine experimental model of aortic banding and heart failure. J Pharmacol Exp Ther. 2005;312(3):891‐898. 15523000 10.1124/jpet.104.077164

[232] Schott P , Singer SS , Kögler H , et al. Pressure overload and neurohumoral activation differentially affect the myocardial proteome. Proteomics. 2005;5(5):1372‐1381. 15732135 10.1002/pmic.200401005

[233] Bupha‐Intr T , Holmes JW , Janssen PML . Induction of hypertrophy in vitro by mechanical loading in adult rabbit myocardium. Am J Physiol Heart Circ Physiol. 2007;293(6):H3759‐H3767. 17933962 10.1152/ajpheart.01267.2006

[234] Ozaki K , Sato H , Inoue K , et al. SNPs in BRAP associated with risk of myocardial infarction in Asian populations. Nat Genetics. 2009;41(3):329‐333. 19198608 10.1038/ng.326

[235] Cao Y , Pan C , Wang YC , et al. Identification of DNA damage repair enzyme Ascc2 as causal for heart failure with preserved ejection fraction. Circulation. 2022;145(14):1102‐1104. 35377742 10.1161/CIRCULATIONAHA.121.055857 PMC8988871

[236] Azqueta A , Collins A . Polyphenols and DNA damage: a mixed blessing. Nutrients. 2016;8(12):E785. 10.3390/nu8120785 PMC5188440 27918471

[237] Serino A , Salazar G . Protective role of polyphenols against vascular inflammation, aging and cardiovascular disease. Nutrients. 2018;11(1):53. 30597847 10.3390/nu11010053 PMC6357531

[238] Münzel T , Gori T , Bruno RM , Taddei S . Is oxidative stress a therapeutic target in cardiovascular disease? Eur Heart J. 2010;31(22):2741‐2748. 20974801 10.1093/eurheartj/ehq396

[239] Dietrich M , Jacques PF , Pencina MJ , et al. Vitamin E supplement use and the incidence of cardiovascular disease and all‐cause mortality in the framingham heart study: does the underlying health status play a role? Atherosclerosis. 2009;205(2):549‐553. 19195657 10.1016/j.atherosclerosis.2008.12.019 PMC2717181

[240] Katsiki N , Manes C . Is there a role for supplemented antioxidants in the prevention of atherosclerosis? Clin Nutr. 2009;28(1):3‐9. 19042058 10.1016/j.clnu.2008.10.011

[241] Mahmoudi M , Gorenne I , Mercer J , Figg N , Littlewood T , Bennett M . Statins use a novel Nijmegen breakage syndrome‐1‐dependent pathway to accelerate DNA repair in vascular smooth muscle cells. Circ Res. 2008;103(7):717‐725. 18723444 10.1161/CIRCRESAHA.108.182899

[242] Pernice F , Floccari F , Caccamo C , et al. Chromosomal damage and atherosclerosis. A protective effect from simvastatin. Eur J Pharmacol. 2006;532(3):223‐229. 16483569 10.1016/j.ejphar.2006.01.003

[243] Aydin S , Uzun H , Sozer V , Altug T . Effects of atorvastatin therapy on protein oxidation and oxidative DNA damage in hypercholesterolemic rabbits. Pharmacol Res. 2009;59(4):242‐247. 19429465 10.1016/j.phrs.2009.01.004

[244] Donmez‐Altuntas H , Bayram F , Coskun‐Demirkalp AN , Baspınar O , Kocer D , Toth PP. Therapeutic effects of statins on chromosomal DNA damage of dyslipidemic patients. Exp Biol Med 2019 244(13):1089‐1095. 10.1177/1535370219871895 PMC6775566 31426681

[245] Vilahur G , Casani L , Peña E , et al. HMG‐CoA reductase inhibition prior reperfusion improves reparative fibrosis post‐myocardial infarction in a preclinical experimental model. Int J Cardiol. 2014;175(3):528‐538. 25023790 10.1016/j.ijcard.2014.06.040

[246] Yoshida M , Shiojima I , Ikeda H , Komuro I . Chronic doxorubicin cardiotoxicity is mediated by oxidative DNA damage‐ATM‐p53‐apoptosis pathway and attenuated by pitavastatin through the inhibition of Rac1 activity. J Mol Cell Cardiol. 2009;47(5):698‐705. 19660469 10.1016/j.yjmcc.2009.07.024

[247] Nübel T , Damrot J , Roos WP , Kaina B , Fritz G . Lovastatin protects human endothelial cells from killing by ionizing radiation without impairing induction and repair of DNA double‐strand breaks. Clin Cancer Res. 2006;12(3 Pt 1):933‐939. 16467108 10.1158/1078-0432.CCR-05-1903

[248] Kim S , Iwao H . Molecular and cellular mechanisms of angiotensin II‐mediated cardiovascular and renal diseases. Pharmacol Rev. 2000;52(1):11‐34. 10699153

[249] Herbert KE , Mistry Y , Hastings R , Poolman T , Niklason L , Williams B . Angiotensin II‐mediated oxidative DNA damage accelerates cellular senescence in cultured human vascular smooth muscle cells via telomere‐dependent and independent pathways. Circ Res. 2008;102(2):201‐208. 17991883 10.1161/CIRCRESAHA.107.158626 PMC2861985

[250] Abdel‐Wahab BA , Metwally ME , El‐khawanki MM , Hashim AM . Protective effect of captopril against clozapine‐induced myocarditis in rats: role of oxidative stress, proinflammatory cytokines and DNA damage. Chem Biol Interact. 2014;5(216):43‐52. 10.1016/j.cbi.2014.03.012 24709159

[251] Khaper N , Singal PK . Modulation of oxidative stress by a selective inhibition of angiotensin II type 1 receptors in MI rats. J Am Coll Cardiol. 2001;37(5):1461‐1466. 11300462 10.1016/s0735-1097(01)01126-3

[252] Keles MS , Bayir Y , Suleyman H , Halici Z . Investigation of effects of Lacidipine, Ramipril and Valsartan on DNA damage and oxidative stress occurred in acute and chronic periods following isoproterenol‐induced myocardial infarct in rats. Mol Cell Biochem. 2009;328(1‐2):109‐117. 19296206 10.1007/s11010-009-0080-y

[253] Brand S , Amann K , Mandel P , Zimnol A , Schupp N . Oxidative DNA damage in kidneys and heart of hypertensive mice is prevented by blocking angiotensin II and aldosterone receptors. PLoS One. 2014;9(12):e115715. 25551569 10.1371/journal.pone.0115715 PMC4297153

[254] Kono Y , Nakamura K , Kimura H , et al. Elevated levels of oxidative DNA damage in serum and myocardium of patients with heart failure. Circ J. 2006;70(8):1001‐1005. 16864932 10.1253/circj.70.1001

[255] Chen YL , Chung SY , Chai HT , et al. Early administration of carvedilol protected against doxorubicin‐induced cardiomyopathy. J Pharmacol Exp Ther. 2015;355(3):516‐527. 26511374 10.1124/jpet.115.225375

[256] Lee J , Lee M , Kim JU , Song KI , Choi YS , Cheong SS . Carvedilol reduces plasma 8‐hydroxy‐2'‐deoxyguanosine in mild to moderate hypertension: a pilot study. Hypertension. 2005;45(5):986‐990. 15837835 10.1161/01.HYP.0000164569.63160.24

[257] Liang ES , Bai WW , Wang H , et al. PARP‐1 (Poly[ADP‐Ribose] Polymerase 1) inhibition protects from Ang II (Angiotensin II)‐induced abdominal aortic aneurysm in mice. Hypertension. 2018;72(5):1189‐1199. 30354818 10.1161/HYPERTENSIONAHA.118.11184

[258] Zingarelli B , Salzman AL , Szabó C . Genetic disruption of poly (ADP‐ribose) synthetase inhibits the expression of P‐selectin and intercellular adhesion molecule‐1 in myocardial ischemia/reperfusion injury. Circ Res. 1998;83(1):85‐94. 9670921 10.1161/01.res.83.1.85

[259] Zingarelli B , Cuzzocrea S , Zsengellér Z , Salzman AL , Szabó C . Protection against myocardial ischemia and reperfusion injury by 3‐aminobenzamide, an inhibitor of poly (ADP‐ribose) synthetase. Cardiovasc Res. 1997;36(2):205‐215. 9463632 10.1016/s0008-6363(97)00137-5

[260] Roesner JP , Mersmann J , Bergt S , et al. Therapeutic injection of PARP inhibitor INO‐1001 preserves cardiac function in porcine myocardial ischemia and reperfusion without reducing infarct size. Shock. 2010;33(5):507‐512. 20395771 10.1097/SHK.0b013e3181c4fb08

[261] Morrow DA , Brickman CM , Murphy SA , et al. A randomized, placebo‐controlled trial to evaluate the tolerability, safety, pharmacokinetics, and pharmacodynamics of a potent inhibitor of poly(ADP‐ribose) polymerase (INO‐1001) in patients with ST‐elevation myocardial infarction undergoing primary percutaneous coronary intervention: results of the TIMI 37 trial. J Thromb Thrombolysis. 2009;27(4):359‐364. 18535785 10.1007/s11239-008-0230-1

[262] Kassan M , Choi SK , Galán M , et al. Enhanced NF‐κB activity impairs vascular function through PARP‐1‐, SP‐1‐, and COX‐2‐dependent mechanisms in type 2 diabetes. Diabetes. 2013;62(6):2078‐2087. 23349490 10.2337/db12-1374 PMC3661639

[263] Nakada Y , Nhi Nguyen NU , Xiao F , et al. DNA damage response mediates pressure overload‐induced cardiomyocyte hypertrophy. Circulation. 2019;139(9):1237‐1239. 30802166 10.1161/CIRCULATIONAHA.118.034822 PMC6467068

[264] Foster CR , Daniel LL , Daniels CR , Dalal S , Singh M , Singh K . Deficiency of ataxia telangiectasia mutated kinase modulates cardiac remodeling following myocardial infarction: involvement in fibrosis and apoptosis. PLoS One. 2013;8(12):e83513. 24358288 10.1371/journal.pone.0083513 PMC3865210

[265] Daniel LL , Daniels CR , Harirforoosh S , Foster CR , Singh M , Singh K . Deficiency of ataxia telangiectasia mutated kinase delays inflammatory response in the heart following myocardial infarction. J Am Heart Assoc. 2014;3(6):e001286. 25520329 10.1161/JAHA.114.001286 PMC4338722

[266] Chiang MH , Liang CJ , Lin LC , et al. miR‐26a attenuates cardiac apoptosis and fibrosis by targeting ataxia–telangiectasia mutated in myocardial infarction. J Cell Physiol. 2020;235(9):6085‐6102. 31990056 10.1002/jcp.29537

[267] Yu H , Zhang F , Yan P , et al. LARP7 protects against heart failure by enhancing mitochondrial biogenesis. Circulation. 2021;143(20):2007‐2022. 33663221 10.1161/CIRCULATIONAHA.120.050812

[268] Thrasher PR , Scofield SLC , Dalal S , Crawford CC , Singh M , Singh K . Ataxia telangiectasia mutated kinase deficiency impairs the autophagic response early during myocardial infarction. Am J Physiol Heart Circ Physiol. 2018;315(1):H48‐H57. 29652546 10.1152/ajpheart.00042.2018 PMC6087781

[269] Jia L , Zhang W , Ma Y , et al. Haplodeficiency of ataxia telangiectasia mutated accelerates heart failure after myocardial infarction. J Am Heart Assoc. 2017;6(7):e006349. 28724653 10.1161/JAHA.117.006349 PMC5586323

[270] Pal S , Nixon BR , Glennon MS , et al. Replication stress response modifies sarcomeric cardiomyopathy remodeling. J Am Heart Assoc. 2021;10(15):e021768. 34323119 10.1161/JAHA.121.021768 PMC8475701

[271] Zhou H , Toan S , Zhu P , Wang J , Ren J , Zhang Y . DNA‐PKcs promotes cardiac ischemia reperfusion injury through mitigating BI‐1‐governed mitochondrial homeostasis. Basic Res Cardiol. 2020;115(2):11. 31919590 10.1007/s00395-019-0773-7

[272] Medunjanin S , Daniel JM , Weinert S , et al. DNA‐dependent protein kinase (DNA‐PK) permits vascular smooth muscle cell proliferation through phosphorylation of the orphan nuclear receptor NOR1. Cardiovasc Res. 2015;106(3):488‐497. 25852083 10.1093/cvr/cvv126

[273] Bartunek J , Vanderheyden M , Knaapen MW , Tack W , Kockx MM , Goethals M . Deoxyribonucleic acid damage/repair proteins are elevated in the failing human myocardium due to idiopathic dilated cardiomyopathy. J Am Coll Cardiol. 2002;40(6):1097‐1103. discussion 1104‐5. 12354434 10.1016/s0735-1097(02)02122-8

[274] Bourgeois A , Bonnet S , Breuils‐Bonnet S , et al. Inhibition of CHK 1 (Checkpoint Kinase 1) elicits therapeutic effects in pulmonary arterial hypertension. Arterioscler Thromb Vasc Biol. 2019;39(8):1667‐1681. 31092016 10.1161/ATVBAHA.119.312537 PMC6727643

[275] Oh H , Wang SC , Prahash A , et al. Telomere attrition and Chk2 activation in human heart failure. Proc Natl Acad Sci USA. 2003;100(9):5378‐5383. 12702777 10.1073/pnas.0836098100 PMC154353

[276] Satoh K , Kikuchi N , Kurosawa R , Shimokawa H . Checkpoint kinase 1 promotes the development of pulmonary arterial hypertension. Arterioscler Thromb Vasc Biol. 2019;39(8):1504‐1506. 31339778 10.1161/ATVBAHA.119.312969

[277] Men H , Cai H , Cheng Q , et al. The regulatory roles of p53 in cardiovascular health and disease. Cell Mol Life Sci. 2021;78(5):2001‐2018. 33179140 10.1007/s00018-020-03694-6 PMC11073000

[278] Mayer DK , Nekhlyudov L , Snyder CF , Merrill JK , Wollins DS , Shulman LN . American Society of Clinical Oncology clinical expert statement on cancer survivorship care planning. J Oncol Pract. 2014;10(6):345‐351. 25316025 10.1200/JOP.2014.001321

[279] Al‐Qasem AJ , Toulimat M , Eldali AM , et al. TP53 genetic alterations in Arab breast cancer patients: Novel mutations, pattern and distribution. Oncol Lett. 2011;2(2):363‐369. 22866089 10.3892/ol.2011.236 PMC3410563

[280] Younes N , Zayed H . Genetic epidemiology of ovarian cancer in the 22 Arab countries: a systematic review. Gene. 2019;5(684):154‐164. 10.1016/j.gene.2018.10.044 30352249

[281] Alhuqail AJ , Alzahrani A , Almubarak H , et al. High prevalence of deleterious BRCA1 and BRCA2 germline mutations in arab breast and ovarian cancer patients. Breast Cancer Res Treat. 2018;168(3):695‐702. 29297111 10.1007/s10549-017-4635-4

[282] El Saghir NS , Zgheib NK , Assi HA , et al. BRCA1 and BRCA2 mutations in ethnic Lebanese Arab women with high hereditary risk breast cancer. Oncologist. 2015;20(4):357‐364. 25777348 10.1634/theoncologist.2014-0364 PMC4391767

